# Contact inhibition controls cell survival and proliferation via YAP/TAZ-autophagy axis

**DOI:** 10.1038/s41467-018-05388-x

**Published:** 2018-07-27

**Authors:** Mariana Pavel, Maurizio Renna, So Jung Park, Fiona M. Menzies, Thomas Ricketts, Jens Füllgrabe, Avraham Ashkenazi, Rebecca A. Frake, Alejandro Carnicer Lombarte, Carla F. Bento, Kristian Franze, David C. Rubinsztein

**Affiliations:** 1 Department of Medical Genetics, Cambridge Institute for Medical Research, Wellcome Trust/MRC Building, Cambridge Biomedical Campus, Hills Road, Cambridge, CB2 0XY UK; 2 Department of Immunology, “Grigore T. Popa” University of Medicine and Pharmacy, Iasi, 700115 Romania; 3 Department of Molecular Medicine and Medical Biotechnology, University of Naples “Federico II”, Naples, 80131 Italy; 4 Department of Physiology Development and Neuroscience, University of Cambridge, CB2 3DY Cambridge, UK; 5 UK Dementia Research Institute, Cambridge Biomedical Campus, Hills Road, Cambridge, CB2 0AH UK

## Abstract

Contact inhibition enables noncancerous cells to cease proliferation and growth when they contact each other. This characteristic is lost when cells undergo malignant transformation, leading to uncontrolled proliferation and solid tumor formation. Here we report that autophagy is compromised in contact-inhibited cells in 2D or 3D-soft extracellular matrix cultures. In such cells, YAP/TAZ fail to co-transcriptionally regulate the expression of myosin-II genes, resulting in the loss of F-actin stress fibers, which impairs autophagosome formation. The decreased proliferation resulting from contact inhibition is partly autophagy-dependent, as is their increased sensitivity to hypoxia and glucose starvation. These findings define how mechanically repressed YAP/TAZ activity impacts autophagy to contribute to core phenotypes resulting from high cell confluence that are lost in various cancers.

## Abstract (web summary)

At high cell density or when plated on soft matrix, YAP/TAZ are redistributed from the nucleus to the cytosol, becoming transcriptionally inactive. Here the authors show that at high cell density, autophagosome formation is impaired due to reduced YAP/TAZ-dependent transcription of actomyosin genes

## Introduction

High-cell density/confluency leads to contact inhibition of proliferation (CIP), a fundamental property whereby normal cells cease proliferation and cell division when they occupy all the space allocated to them upon reaching confluence^1^. This arrest of cell proliferation is seen in most epithelial cells, and is associated with a halt in cell division and the initiation of differentiation. CIP is reversed in physiological conditions requiring rapid cell growth and proliferation, such as embryonic development and wound healing or tissue regeneration. Pathologically, loss of contact inhibition leads to uncontrolled cell growth (characteristic of solid tumors) and increases the abilities of cells to invade host tissues (as in metastasis)^2–4^.

The mechanism behind these mechanical signals (of contact inhibition or cell shape deformation generated by the pulling forces of the ECM) has only recently been linked to Hippo signaling^5–7^, a pathway comprising two interconnected core modules: kinases (MST1/2, LATS1/2 kinases) and transcriptional regulators (YAP/TAZ co-transcriptional regulators and TEADs transcription factors). When cells are at low density and are flat/well-spread on a stiff extracellular matrix (ECM), YAP/TAZ localize in the nucleus and are active, while when the cells are round/compact at high-cell density or plated on soft matrix with minimum adhesion area to the ECM, YAP/TAZ are redistributed to the cytosol and are inactive^7–9^. As Hippo signaling impacts cancer initiation/progression, organ development, and stem cell maintenance and regeneration^10–13^, it is important to understand relevant effector processes downstream of YAP/TAZ, as cell proliferation and survival. Autophagy is also a key player in assisting cell survival during nutrients or oxygen deprivation conditions, important stresses associated with cancerous environments^14,15^.

Here we show that YAP and TAZ promote autophagy thorough transcriptional regulation of myosin-II and conversely, autophagy is crucial in maintaining both the cell survival and proliferative status downstream of the Hippo signaling hubs, YAP/TAZ–TEAD.

## Results

### Autophagosome formation is reduced at high cell density

**Fig. 1:**
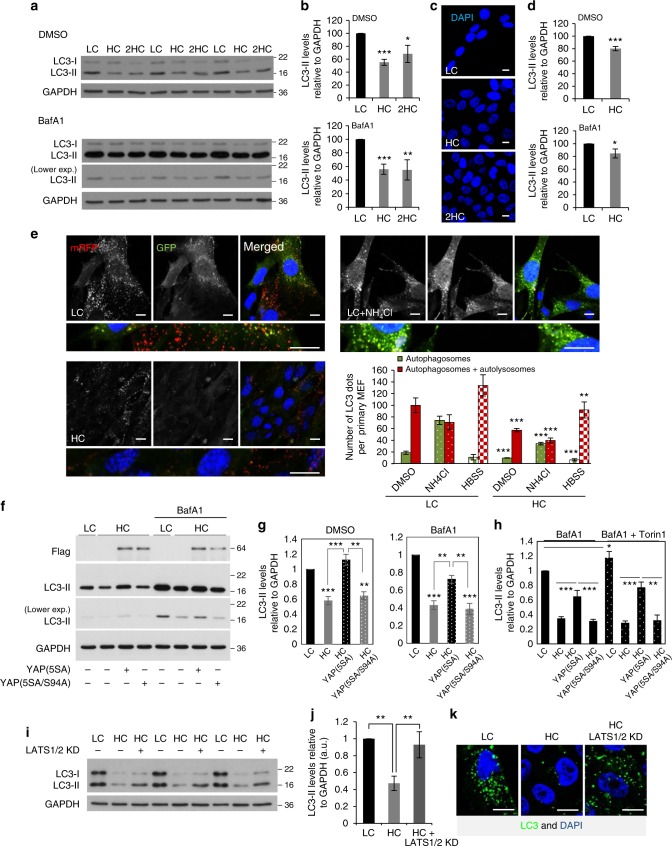
Autophagosome formation is reduced at high cell density via YAP/TAZ inhibition. **a** LC3-II levels assessed by immunoblotting in MCF10A cells plated at different confluencies: LC (low confluency) and HC (high confluency). 2HC – twice as many cells plated as in HC. The cells were treated with vehicle (DMSO) or bafilomycin A1 (BafA1) at 400 nM for 5 h. GAPDH was used as loading control. **b** Densitometry of LC3-II/GAPDH blots obtained from MCF10A cells plated at different confluencies as in **a**. The graphs show the mean ± s.d. (*n* = 3; ***P < 0.001, **P < 0.01, *P < 0.05; two-tailed one sample *t*-test). **c** Representative images of DAPI staining at different confluencies of MCF10A cells, used in **a**, **b**. Scale bar is 10 µm. **d** Densitometry of LC3-II/ Gapdh levels in primary MEFs plated at different confluencies. The graphs show the mean ± s.d. (*n* = 3; ***P < 0.001, *P < 0.05; two-tailed one sample *t*-test). **e** Representative confocal images (see left panel) and total number of GFP dots (autophagosomes) and mRFP dots (autophagosomes and autolysosomes)—right panel, in primary MEFs from transgenic mice expressing mRFP-GFP-LC3. MEFs plated at LC and HC were fixed and subjected to confocal visualization. The numbers of dots were counted automatically using a Cellomics microscope (*n* = 4; ***P < 0.001, **P < 0.01; two-tailed *t*-test). More than 600 cells were counted per experiment, per condition. Scale bar is 10 µm. **f** LC3-II levels assessed by immunoblotting in HeLa cells plated at different confluencies and overexpressing control (Ctrl), YAP(5SA) or YAP(5SA/S94A). These cells were exposed to either control (DMSO) or BafA1 (400 nM), for the last 5 h. GAPDH was used as loading control. **g** LC3-II/GAPDH densitometry of HeLa cells treated as in **f**. The graphs show the mean ± s.e.m. (*n* = 6; ***P < 0.001, **P < 0.01; two-tailed one sample *t*-test). **h** LC3-II/GAPDH densitometry of HeLa cells overexpressing either YAP(5SA) or YAP(5SA/S94A) in the presence of BafA1 and/or Torin1 (used as mTORC1 inhibitor). GAPDH was used as loading control. Bars represent the mean ± s.d. (*n* = 3; ***P < 0.001, **P < 0.01, *P < 0.1; two-tailed *t*-test). **i** LC3-II levels assessed by immunoblotting in MCF10A cells plated at different confluencies and exposed to either control (Ctrl) or LATS1/2 siRNAs. GAPDH was used as loading control. **j** LC3-II/GAPDH densitometry of MCF10A treated as in **h**. The graphs show the mean ± s.d. (*n* = 4; **P < 0.01; two-tailed one sample *t*-test). **k** Representative images of endogenous imunostaining of LC3 in MCF10A cells treated as above. Quantification of number of LC3 dots per cell and enlarged images are presented in Supplementary Fig. 4b. *n* = number of independent biological replicates unless otherwise stated

We noticed that isolated or well-spread out (“sparse”) MCF10A cells (non-tumorigenic epithelial cells) on coverslips had more LC3 endogenous puncta (autophagosomes), compared to the cells in the middle of confluent cell patches—described here as “dense” (Supplementary Fig. 1a). In densely populated cells, the perinuclear pool of LC3 was significantly reduced by at least 50% (Supplementary Fig. 1a), while the pool of LC3 in close proximity to the plasma membrane/cell periphery was still prominent. We confirmed the inverse relationship between cell density and autophagosome number by examining cells at (a) low confluency (or low density or sparsity), when the cells were seeded in such a way that they had minimal or no contact with neighboring cells, (b) confluent, where all the cells had some degree of contact with neighboring cells (an intermediate/transition stage between low confluency and high confluency), and (c) high confluency (or high density), when cells were cultured to occupy all the allocated space in a dense and compact monolayer, a cell density state highly associated with contact inhibition of proliferation. In MCF10A (Fig. 1a–c), HeLa (Supplementary Fig. 1b), HaCaT cells (Supplementary Fig. 1c), and in primary mouse embryonic fibroblasts (pMEFs) (Fig. 1d and Supplementary Fig. 1d), LC3-II levels (which correlate with autophagosome load) were significantly reduced at high cell confluency. This phenomenon was also seen in the presence of bafilomycin A1 (Baf A1), which blocks LC3-II/autophagosome degradation, allowing one to infer that high confluency inhibits LC3-II/autophagosome formation^16^ (Fig. 1a–d and Supplementary Fig. 1b–d). The LC3-II levels were not further reduced even when we plated twice as many cells (named “2HC”) than in the high cell confluency (HC) condition, suggesting that autophagosome formation is regulated by cell density only until a certain cell confluency is reached, and not by the cell size per se (Fig. 1a–c and Supplementary Fig. 1f).

In primary MEFs (Fig. 1e) and primary mammary epithelial cells (pMECs) (Supplementary Fig. 1g–h) from mice transgenically expressing mRFP-GFP-LC3^17^ and in HeLa cells stably expressing mRFP-GFP-LC3 reporter (Supplementary Fig. 1i), cells at high confluency showed reduced numbers of autophagosomes (both GFP- and mRFP-positive) and autolysosomes (mRFP-only positive, due to quenching of the GFP by the lysosomal pH^18,19^). Decreased autophagosome numbers were also observed in high confluency cells when these were either starved (as an autophagy stimulus; HBSS) or treated with NH_4_Cl (inhibitor of lysosomal acidification), as shown by the quantification of autophagosome numbers in primary MEFs in Fig. 1e. Thus, high cell confluency inhibits autophagosome biogenesis in both basal and autophagy-induced conditions.

As expected, the levels of the well-characterized autophagy substrate, p62^20,21^ were increased in high confluent MCF10A cells, reinforcing the autophagy inhibition phenotype seen under such conditions (Supplementary Fig. 2a). High confluency cells also showed increased accumulation of two additional autophagy substrates: mutant huntingtin exon 1 tagged to GFP in HeLa cells^17,22^ (where levels of mutant huntington (htt) exon 1 correlate with the percentage of cells with aggregates^23^, thus the percentage of cells with N17-97QP-GFP aggregates correlate with autophagic activity^17^—Supplementary Fig. 2b), and soluble A53T-synuclein tagged to GFP^24^ (the ratio between soluble A53T-syn GFP and empty-GFP) in both MCF10A (Supplementary Fig. 2c) and HeLa cells (Supplementary Fig. 2d).

Clearly, from a practical perspective, these results highlights that one should be vigilant to changes in cell confluency being a cause of altered autophagy in tissue culture experiments, as this may arise from genetic or chemical perturbations to cell growth or through imprecise seeding.

The reduced autophagy in highly confluent cells is not due to mTOR hyperactivation or AMPK inhibition, as high confluency cells have reduced phosphorylated p70-S6K (mTORC1 substrate) and phospho-AKT (S473) (mTORC2 substrate)—Supplementary Fig. 2e–f, and increased phospho-ULK1(S555) (AMPK substrate)—Supplementary Fig. 2g, as previously reported^25–28^, and high confluency compromised autophagy in starved cells where mTORC1 is inhibited (Fig. 1e).

### YAP/TAZ double knockdown inhibits autophagosome formation

As expected, YAP/TAZ were predominantly cytoplasmic (inactive) in high confluent MCF10A cells (both HC and 2HC conditions), compared to low confluency (LC) or confluent (C) states where YAP/TAZ are mainly nuclear (active) or present in both nucleus and cytosol and the amount of nuclear YAP/TAZ correlated with the cell proliferative status indicated by BrdU staining (Supplementary Fig. 3a; for additional cell types – Supplementary Fig. 3b–e).

Thus, we tested if YAP/TAZ loss-of-function (mimicking HC) impaired autophagosome biogenesis in low confluent conditions and whether YAP/TAZ reactivation in high confluent state could rescue the autophagy inhibition phenotype. Since YAP and TAZ act as co-regulators of TEAD transcription factors and are therefore largely redundant^11^, knockdown of either YAP or TAZ does not phenocopy HC (Supplementary Fig. 4a, b). Double YAP/TAZ knockdown significantly reduced the LC3-II levels/puncta in the absence and presence of Baf A1 in both MCF10A (Supplementary Fig. 4c) and HeLa cells using both smart-pool (Supplementary Fig. 4d–e) and individual siRNA oligos for different time points (96 h knockdown—Supplementary Fig. 4f, and 36 h knockdown—Supplementary Fig. 4g). Likewise, double YAP/TAZ siRNA knockdown decreased autophagosome numbers in HeLa cells stably expressing GFP-LC3 (Supplementary Fig. 4e) and similar effects were seen when we treated low confluency cells with verteporfin, which inhibits YAP/TAZ co-transcriptional activity (Supplementary Fig. 4h-j). As expected, verteporfin had no effect on autophagy in high confluency cells (Supplementary Fig. 4k). Consistent with these data, miR-375 (a well-characterized micro-RNA targeting YAP^29^) also reduced LC3-II synthesis in MCF10A cells (Supplementary Fig. 4l). Thus, YAP/TAZ inhibition reduced autophagosome biogenesis and may explain the autophagy compromise seen in high confluent cells, a possibility strengthened by the observation that overexpression of constitutively active YAP(5SA), but not inactive YAP(5SA/S94A), rescued the autophagy inhibition observed in high confluency cells, both in the presence and absence of BafA1 (Fig. 1f–h and Supplementary Fig. 5a). In addition, YAP5(SA), but not YAP(5SA/S94A), reactivated mTOR in HC (Supplementary Fig. 5b). Interestingly, the YAP(5SA) effect on autophagy induction in HC cells was also seen in the presence of Torin 1 (a well characterized potent mTOR inhibitor)—Supplementary Fig. 5a, suggesting a mechanism independent of mTOR activity.

Hippo signaling acts through the activation of LATS1/2 kinases, which phosphorylate and, thereby, inactivate YAP and TAZ^5,30^. Phosphorylated YAP and TAZ are then sequestered in the cytosol by various proteins^31–33^. As inactivation of the LATS1/2 kinases in high cell density conditions rescues the nuclear localization of YAP/TAZ (confirmed in Supplementary Fig. 5c) and their activity^34,35^, we used this strategy to confirm that the autophagy inhibition in HC was YAP/TAZ-dependent. High confluency cells exposed to LATS1/2 siRNAs showed increased levels of LC3-II (Fig. 1i, j) or autophagosome numbers (Fig. 1k and Supplementary Fig. 5d) compared to control HC cells. The rescue of autophagy mediated by LATS1/2 KD in HC was YAP/TAZ-dependent, as this was abrogated by YAP/TAZ double knockdown (Supplementary Fig. 5e, f). The overexpression of the MST2 kinase which is responsible for the phosphorylation and activation of the LATS1/2 kinases also reduced the LC3-II levels both in the absence or presence of BafA1 (Supplementary Fig. 5g). MST1/2 directly phosphorylates LC3 (at Thr^36^), which is critical for the proper fusion of autophagosomes with lysosomes^37^. However, our data argue that a key role for MST1/2 overexpression is at the level of autophagosome biogenesis.

### YAP/TAZ transcriptionally modulate the F-actin cytoskeleton

As YAP and TAZ are co-transcriptional regulators, we next analyzed published ChIP-Seq data (see Methods) to explore how they may regulate autophagy (Supplementary Fig. 6a). Although we identified a group of autophagy-related genes containing ULK1, ATG16L1, ATG9, and ATG3, qPCR array analyses did not validate those genes as real targets of YAP/TAZ (Supplementary Table 1).

**Fig. 2:**
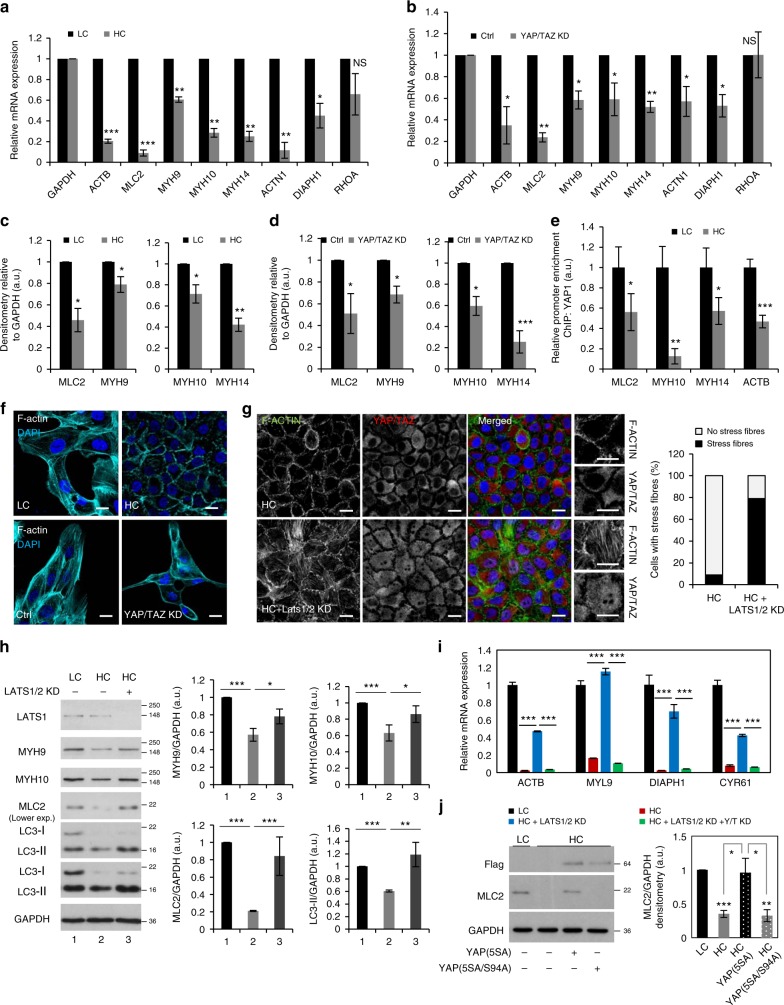
High confluent cells show dramatic reduction of stress fibers formation and have low expression of actomyosin proteins via YAP/TAZ inhibition. **a** mRNA levels of the indicated proteins relative to GAPDH (control) in MCF10A cells plated at different confluencies: LC and HC. **b** mRNA levels of the indicated proteins relative to GAPDH (control) in MCF10A cells treated with YAP/TAZ siRNAs. **c** Densitometry of the indicated protein levels relative to GAPDH (control) in MCF10A cells plated at different confluencies: LC and HC. **d** Densitometry of the indicated protein levels relative to GAPDH (control) in MCF10A cells treated with YAP/TAZ siRNAs. **e** Relative promoter enrichment in YAP of the indicated genes. MCF10A cells were seeded at LC and HC and subjected to ChIP:YAP analysis. Bars represent the mean ± s.d. of one representative experiment performed in triplicates (***P < 0.001, **P < 0.01, *P < 0.05; two-tailed *t*-test). The experiment was repeated at least another 2 times with similar results. **f** Representative images of F-actin (Phalloidin) immunostaining in MCF10A cells plated at low and high confluencies and exposed to either Ctrl or YAP/TAZ double knockdown. Scale bar is 10 µm. **g** Representative images of F-actin (Phalloidin) immunostaining in MCF10A cells plated at high confluency and treated with LATS1/2 siRNAs. The number of cells with stress fibers is rescued by LATS1/2 KD (see right panel). **h** Representative western-blots for the indicated proteins in MCF10A cells plated at high confluency and treated with LATS1/2 siRNAs. The levels of the indicated proteins are rescued by LATS1/2 KD. **i** Relative mRNA levels of the indicated genes to GAPDH in MCF10A cells exposed to LATS1/2 and/or YAP/TAZ siRNAs. The treated cells were seeded at low and high confluencies. Bars represent the mean ± s.d. of one representative experiment performed in triplicates (***P < 0.001; two-tailed *t*-test). **j** MCL2 levels assessed by immunoblotting in HeLa cells plated at different confluencies and overexpressing control (Ctrl), YAP(5SA) or YAP(5SA/S94A). GAPDH was used as loading control. MLC2/GAPDH densitometry is displayed on the right panel. The graphs show the mean ± s.e.m. (*n* = 4; ***P < 0.001, **P < 0.01, *P < 0.05; two-tailed one sample *t*-test). Unless otherwise stated, bars—mean ± s.d. (*n* = 3;***P < 0.001, **P < 0.01, *P < 0.05, NS, not significant; two-tailed one sample *t*-test). *n* = number of independent biological replicates unless otherwise stated

Another cluster of genes closely related to autophagy was composed of actin-related genes involved in the formation of stress-fibers (see Supplementary Fig. 6b). mRNA expression (Fig. 2a, b and Supplementary Fig. 6d–h) and protein levels (Fig. 2c, d and Supplementary Fig. 6i, j) of actin-related genes were lowered by about 50% in both high confluent and YAP/TAZ knockdown cells: MCF10A, primary MEFs and HeLa cells. Consistently, verteporfin treatment also reduced the myosin light chain 2 (MLC2) levels in HeLa cells (Supplementary Fig. 6k). In addition, diaphanous homolog 1 (DIAPH1), which is required for the formation of F-actin structures^38^, and was suggested in previous studies to participate in the early events of autophagosome formation upon starvation^39^, was also reduced in both HC and YAP/TAZ KD conditions (Supplementary Fig. 6j). As expected, classical YAP/TAZ target genes—CYR61, CTGF, ANKRD1, were significantly reduced in HC- compared to LC-MCF10A cells and primary MEFs (Supplementary Fig. 6c, d).

To consolidate these results, we performed chromatin-immunoprecipitation (ChIP) experiments to validate the binding of YAP/TAZ at the promoter regions of some of the selected genes. Because YAP and TAZ act as co-transcriptional regulators mainly by regulating the activity of TEAD transcription factors, we searched for TEAD binding motifs (“CATTCC”, or variations^8,40,41^) in the promoters of ACTB, MLC2, MYH10, and MYH14 (Supplementary Fig. 6l). Binding of YAP/TAZ to TEAD-containing regions in these gene promoters was reduced in high confluent cells (Fig. 2e and Supplementary Fig. 6m, n), confirming that these selected targets are indeed direct targets of YAP/TAZ co-transcriptional regulation.

Consistent with the expression data, F-actin stress fibers were dramatically reduced in both high confluent and YAP/TAZ knockdown cells (Fig. 2f and Supplementary Fig. 7a). To determine whether the loss of stress fibers under HC conditions is YAP/TAZ-dependent, we designed rescue experiments by knocking-down LATS1/2 in high confluent cells, as we previously showed that autophagy inhibition is rescued under these conditions. This was important as LATS1/2 kinases may also affect the F-actin dynamics in a manner independent of YAP/TAZ activity^42–44^. LATS1/2 or LATS1/2 and NF2 double knockdowns rescued F-actin stress fibers in high confluent MCF10A cells fibers (Fig. 2g and Supplementary Figs. 7b–c and 8a–b), but this rescue was lost in cells depleted of YAP/TAZ (Supplementary Fig. 7b, c). These data are in agreement with previous studies that showed that overexpression of constitutively active YAP(5SA), but not YAP(5SA/S94A) induces the formation of F-actin stress fibers in high confluency cells^6^. Indeed, we confirmed these observations also in HeLa cells and found that YAP(5SA) overexpression and not YAP(5SA/S94A) was able to rescue the formation of F-actin stress fibers in HC conditions (Supplementary Fig. 9).

LATS1/2 KD rescued the levels of all the components of the myosin-II complex in HC (MLC2—myosin light chain and MYH9, MYH10, MYH14—myosin heavy chain isoforms) (Fig. 2h) and mRNA levels (Fig. 2i) that were reduced in HC. As expected, the rescue effect on the mRNA levels of the actin cytoskeleton genes by LATS1/2 KD was abolished in cells depleted of YAP/TAZ (Fig. 2i). MLC2 levels also increased upon YAP(5SA) (and not YAP(5SA/S94A)) overexpression under HC conditions (Fig. 2j). Thus, the loss-of F-actin stress fibers seen in HC is dependent on the transcriptional regulation of myosin-II and other actin-related genes by YAP/TAZ.

### Myosin-II is the downstream effector of YAP/TAZ on autophagy

**Fig. 3:**
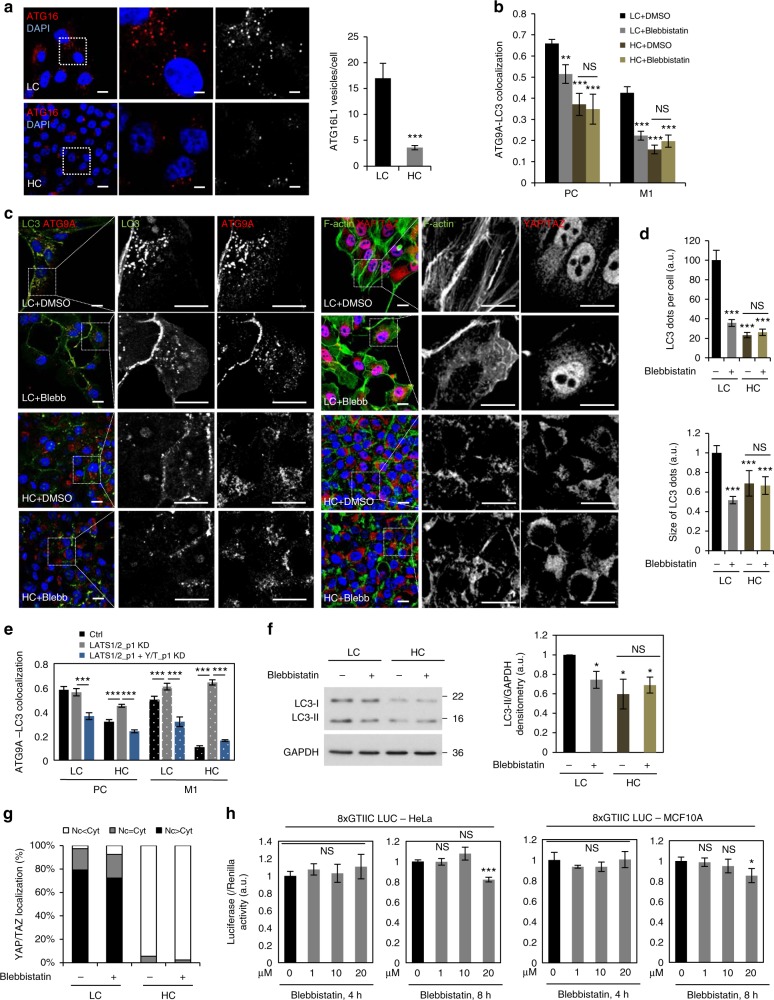
Myosin-II inhibition reduces autophagosome biogenesis in low confluency cells. **a** Representative images of endogenous ATG16L1 vesicles in MCF10A cells. Scale bar is 10 µm. Right panel: quantification of ATG16L1 vesicles numbers in LC and HC. At least 20–30 cells were quantified per condition. **b** ATG9A—LC3 colocalization in MCF10A cells. MCF10A cells plated at low and high confluencies were exposed to either DMSO (vehicle control) or blebbistatin (20 µM) for 4 h. The Pearson’s correlation and Manders’ overlap (the amount of LC3 in the ATG9 compartment) coefficients were used to measure the colocalization of LC3 with ATG9A in >30 cells per condition. **c** Representative confocal images of LC3 - ATG9A and z-stack images of F-actin and YAP/TAZ double immunostainings in MCF10A cells treated as in **b**. Scale bar is 10 µm. **d** Number and size of LC3 dots per cell in MCF10A cells plated at various densities and exposed to either DMSO (vehicle control) or blebbistatin (20 µM) for 4 h. Representative images–see **b**. More than 30 cells were analyzed per condition using ImageJ. **e** ATG9A—LC3 colocalization in MCF10A cells plated at LC or HC confluencies and exposed to either LATS1/2_p1 alone or combined with YAP/TAZ_p1 siRNAs. The Pearson’s correlation and Manders’ overlap (the amount of LC3 in the ATG9 compartment) coefficients were used to measure the colocalization of LC3 with ATG9A (for LC: *n* = 25 cells, while for HC: *n* = 100 cells). See Supplementary Fig. 10 for representative images. **a**–**e** Bars—mean ± s.e.m. (***P < 0.001, **P < 0.01, *P < 0.05, NS, not significant; two-tailed *t*-test). **f** Representative LC3-II immunoblots for MCF10A cells treated with blebbistatin (20 µM, 5 h). LC3-II/ GAPDH densitometry in MCF10A cells exposed to blebbistatin (20 µM, 5 h)–bottom panel. Bars—mean ± s.d. (*P < 0.05, NS, not significant; two-tailed one sample *t*-test). **g** YAP/TAZ localization in MCF10A cells treated as in **b**, **c**. There is no dramatic difference in YAP/TAZ localization between control and blebbistatin-treated cells. **h** Luciferase assay for YAP/TAZ activity in HeLa and MCF10A cells exposed to increasing concentrations of blebbistatin for 4 and 8 h, respectively. Bars–mean ± s.d. (*n* = 3, ***P < 0.001, *P < 0.05, NS, not significant; two-way ANOVA). *n* = number of independent biological replicates unless otherwise stated

The actin cytoskeleton and myosin-II modulate the trafficking of key autophagy proteins, like ATG16L1 and ATG9A, that are required for autophagosome formation^45,46^. HC was associated with a reduction in the numbers of ATG16L1 puncta (autophagosome precursors) per cell (Fig. 3a) and with decreased colocalisation of ATG9A - LC3 and (Fig. 3b–e), a phenomenon that correlates with reduced autophagosome biogenesis^47^. The localization of ATG9A was altered, from preferentially perinuclear in low confluency cells, to scattered in high confluency cells (Fig. 3c), while total ATG9 levels were not reduced in YAP/TAZ depleted cells (see Supplementary Table 1)^48^.

Blebbistatin (a specific myosin inhibitor) caused actin depolymerization (Fig. 3c), reduced ATG9A-LC3 colocalization (Fig. 3b, c) and decreased the number/ size of LC3 puncta (per cell) (Fig. 3d) and the total LC3-II levels (Fig. 3f) in low confluency cells, but failed to further reduce these autophagy parameters in high confluency cells (Fig. 3b–d, f), despite the fact that it did not have an obvious impact on YAP/TAZ cellular localization or transcriptional activity (Fig. 3c, g, h). These data suggest that inhibition of the myosin–II complex and consequent actin depolymerization are sufficient to explain the autophagy inhibition in HC.

**Fig. 4:**
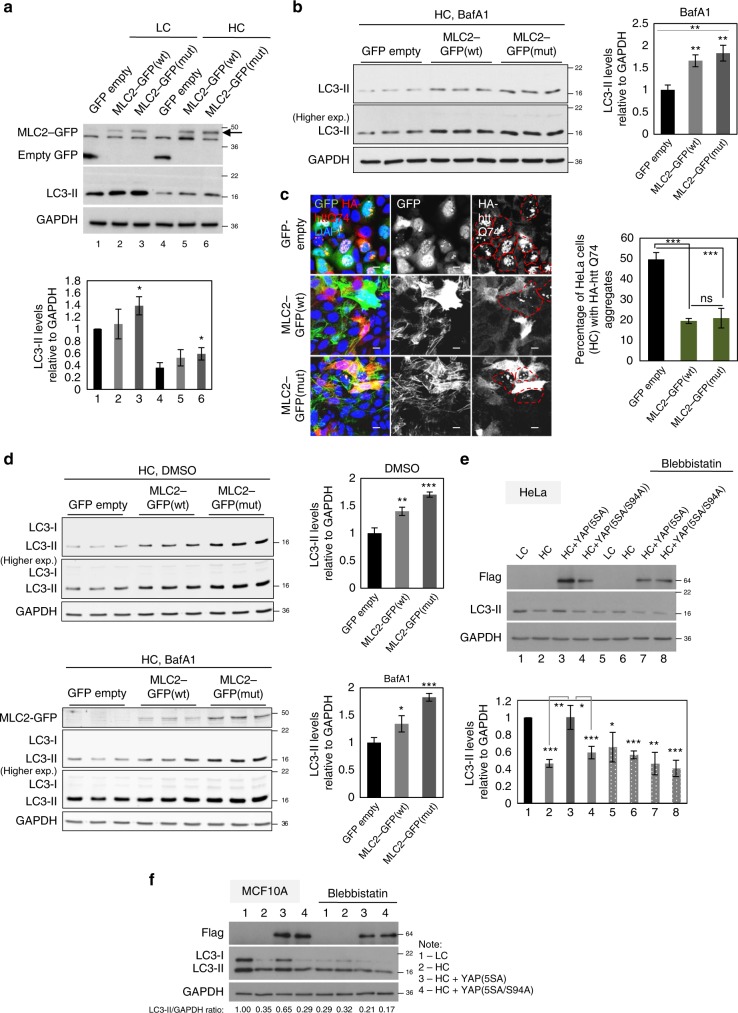
The autophagy inhibition caused by high cell density is myosin II –dependent. **a** Representative LC3-II western-blots of HeLa cells overexpressing either the wild-type or the constitutively active MLC2 (T18D, S19D—mut) construct. The graphs show the LC3-II/GAPDH: means ± s.d. (*n* = 3; *P < 0.05; two-tailed one sample *t*-test). **b** Representative LC3-II western-blots of high confluency HeLa cells overexpressing either the wild-type or the constitutively active MLC2 (T18D, S19D - mut) construct. The cells were exposed to BafA1 (400 nM). The graphs show the LC3-II/GAPDH: means ± s.d. (*n* = 3; **P < 0.01; two-way ANOVA). **c** Representative HA-htt Q74 aggregates in high confluency HeLa cells overexpressing either the wild-type or the constitutively active MLC2 (T18D, S19D - mut) construct. The graphs show the percentage of cells with HA-htt Q74 aggregates: means ± s.d. (*n* = 3; ***P < 0.001, NS, not significant; two-way ANOVA). **d** Representative LC3-II western-blots of high confluency MCF10A cells overexpressing either the wild-type or the constitutively active MLC2 (T18D, S19D - mut) construct. The cells were exposed to either DMSO or BafA1 (400 nM). The graphs show the LC3-II/GAPDH: means ± s.d. (*n* = 3; ***P < 0.001, **P < 0.01, *P < 0.05; two-way ANOVA). **e** Representative LC3-II western-blots of HeLa cells overexpressing control (empty flag), YAP(5SA) or YAP(5SA/S94A) exposed to either vehicle (DMSO) or blebbistatin treatment. Bars represent the mean ± s.d. (*n* = 3; ***P < 0.001, **P < 0.01, *P < 0.05; two-tailed one sample *t*-test). **f** Representative LC3-II western-blots of MCF10A cells overexpressing control (empty flag), YAP(5SA) or YAP(5SA/S94A) exposed to either vehicle (DMSO) or blebbistatin treatment. *n* = number of independent biological replicates unless otherwise stated

LATS1/2 double knockdown using two different pairs of individual siRNA oligos was able to increase the ATG9A-LC3 colocalisation in high confluency cells in a manner dependent on YAP/TAZ activity (Fig. 3e and Supplementary Fig. 10–11). The increase in the amount of LC3 in the ATG9A compartment given by LATS1/2 depletion is also dependent on the myosin-II activity as treatment with blebbistatin of LATS1/2 knockdown high confluency MCF10A cells abolished this effect (Supplementary Fig. 12a, b). Next, overexpressions of either the wild-type or the constitutively active MLC2 (T18D, S19D) were able to double the amount of LC3-II (Fig. 4a, b) and reduce the percentage of cells with HA-htt Q74 aggregates in high confluency HeLa cells (Fig. 4c). Accordingly, overexpression of both forms of MLC2 (wild-type or constitutively active) significantly increased the levels of LC3-II both in the presence and absence of BafA1 in high confluency MCF10A cells (Fig. 4d). Furthermore, the YAP(5SA)-dependent rescue of LC3-II levels under HC was abolished in the presence of the myosin-inhibitor, blebbistatin both in HeLa (Fig. 4e) and MCF10A cells (Fig. 4f).

Thus, we concluded that at high cell density, Hippo signaling is activated and relocalises YAP/TAZ from the nucleus to the cytosol, which leads to reduced YAP/TAZ co-transcriptional regulation of the myosin–II complex and other actin cytoskeleton-related genes. This is followed by complete loss of F-actin stress fibers, impaired trafficking of key autophagy proteins and reduced autophagosome biogenesis. However, we cannot exclude that other YAP/TAZ targets (others than the mentioned actin-related genes) might contribute to the defects in autophagosome biogenesis seen in high confluency cells.

### YAP/TAZ monitor F-actin dynamics downstream of CAPZB

**Fig. 5:**
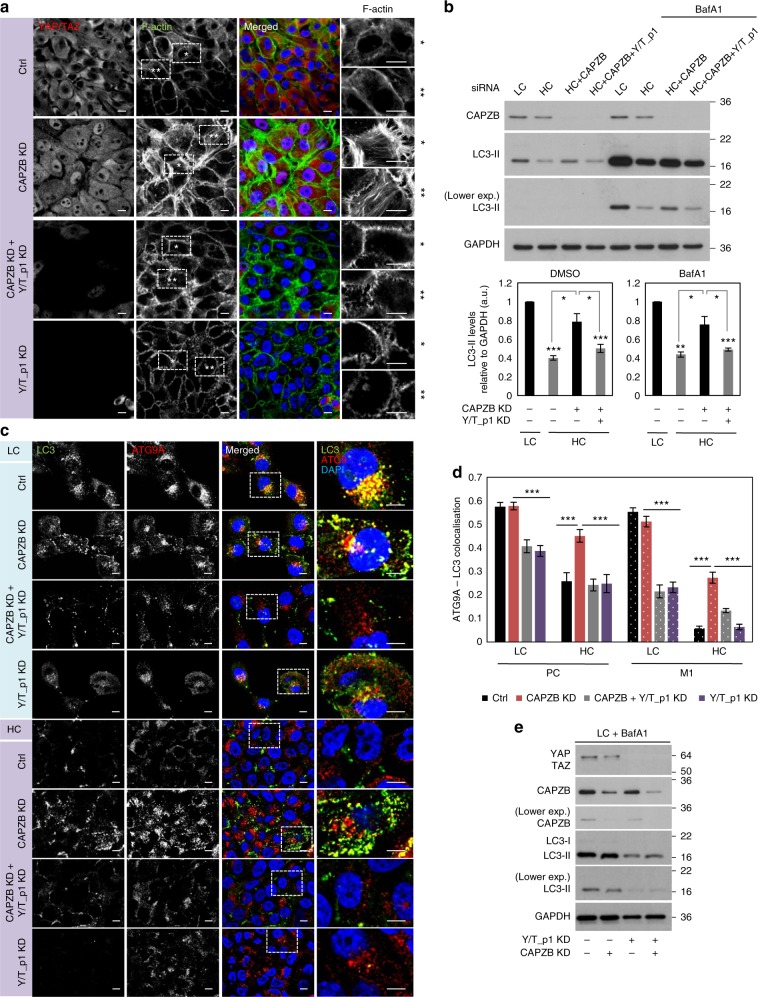
The rescue of F-actin stress fibers by CAPZB KD in HC is YAP/TAZ-dependent. **a** Representative confocal images of MCF10A cells exposed to control, CAPZB or YAP+TAZ (Y/T_p1) siRNA oligos. MCF10A cells were immunostained for endogenous YAP/TAZ and F-actin. Scale bars are 10 µm. **b** Representative LC3-II western-blots of MCF10A cells exposed to either vehicle (DMSO) or blebbistatin treatment. MCF10A cells were transfected with the corresponding siRNAs and plated at low and high confluencies. Bars represent the mean ± s.d. (*n* = 3; ***P < 0.001, **P < 0.01, *P < 0.05; two-tailed one sample *t*-test). **c** Representative images of LC3 and ATG9A double immunostaining in MCF10A cells plates at LC and HC confluencies. MCF10A cells were exposed to CAPZB and/or YAP/TAZ (Y/T_p1) siRNAs and plated at low and high confluencies. Scale bar is 10 µm. **d** Quantification of ATG9A – LC3 colocalization in MCF10A cells plated and treated as in (**c**). The Pearson’s correlation and Manders’ overlap (the amount of LC3 in the ATG9A compartment) coefficients were used to measure the colocalization of LC3 with ATG9A in more than 30 and 100 cells in LC and HC conditions, respectively (***P < 0.001; two-tailed *t*-test). **e** Representative LC3-II western-blots of low confluency MCF10A cells exposed to CAPZB and/or YAP/TAZ (Y/T_p1) siRNAs. *n* = number of independent biological replicates unless otherwise stated

To test the role of the actin cytoskeleton on autophagy in HC further, we depleted an endogenous negative regulator of F-actin, the F-actin-capping protein, CAPZB. In high confluency MCF10A cells, CAPZB knockdown induced YAP/TAZ shuttling into the nucleus and also increased F-actin staining (both F-actin ventral stress fibers and lamellipodia—microspikes) (Fig. 5a). Interestingly, YAP/TAZ depletion markedly diminished this effect on actomyosin cytoskeleton formation (acting mainly on the ventral F-actin stress fibers)—Fig. 5a. In parallel, in HC-MCF10A cells, CAPZB-depletion also enhanced LC3-II lipidation (either in the presence or absence of BafA1; however, to a lesser extent than LATS1/2 depletion), an effect that was diminished when YAP and TAZ were concomitantly knocked down (Fig. 5b). Indeed, in high confluent cells, CAPZB was able to partially rescue the amount of LC3 in the ATG9A compartment to ~50% of the low confluency control value in a YAP/TAZ-dependent manner (Fig. 5c, d). Conversely, in sparse, low confluent cells^49^, CAPZB did not affect LC3-II lipidation per se (Fig. 5e) and LC3-ATG9A colocalisation (Fig. 5c, d). Thus, the YAP/TAZ-dependent transcription of actomyosin genes modulates the F-actin dynamics, which regulates autophagosome formation.

It has also been previously reported that cells detached from the substrate have inhibited YAP/TAZ^36,50–52^. However, in these conditions, when low confluency cells are detached by trypsinisation, autophagy is rather upregulated than inhibited, as our data confirm in both the presence and absence of BafA1 (Supplementary Fig. 13a, b). Interestingly, when high confluency MCF10A cells were detached and kept in suspension for various time points (the experiment was performed in parallel with those low confluency cell detachment), we could not observe any increase in autophagy levels measured by LC3-II levels (Supplementary Fig. 13c). More surprisingly, when cells were detached by scraping, LC3-II levels were reduced both in the presence and absence of BafA1 (Supplementary Fig. 13d), an autophagy phenotype similar to our high confluency conditions. The most likely explanation for these observations is that the high confluency state is different from the full cell detachment, as other factors may be involved in autophagy regulation, like the presence or absence of protein adhesions or the different time points required for achieving the two conditions. As YAP/TAZ depletion phenocopies the high confluency state (in terms of autophagy inhibition), we believe that the main players regulating autophagy in HC are YAP/TAZ. Further, to reinforce the autophagy inhibition in HC cells, as autophagy is required for cell attachment, low and high confluency cells were resuspended and the same number of cells were plated per well, in order to count for the number of cells attached after different time points (4, 8, 12, 24 h)—Supplementary Fig. 14. Indeed, the initial low confluency cells adhered faster to the substrate than their counterparts, the high confluncy cells (Supplementary Fig. 14b).

### Consequences of autophagy inhibition at high cell density

**Fig. 6:**
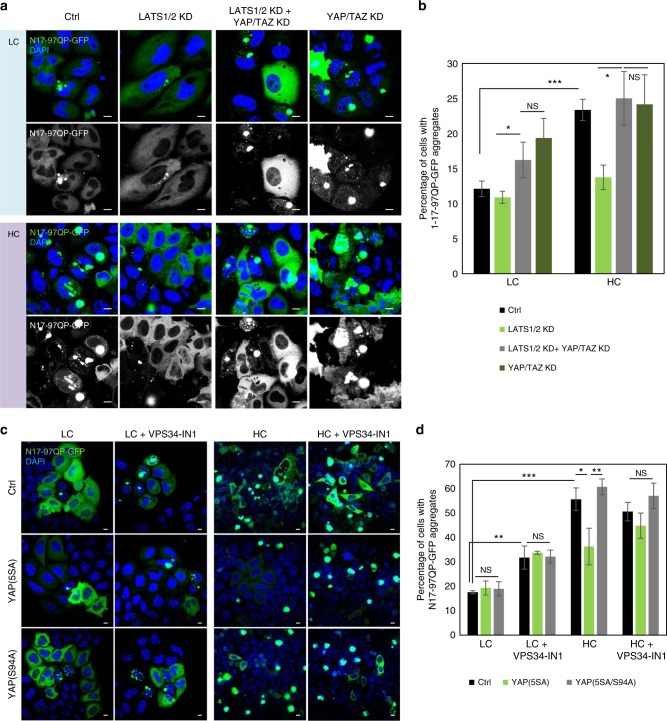
The accumulation of mutant huntingtin in high confluency HeLa cells is YAP/TAZ- and autophagy-dependent. **a** Representative images of N17-97QP-GFP aggregates in HeLa cells treated with LATS1/2 and/or YAP/TAZ siRNAs, transfected with N17-Q97P-GFP (1 µg) and plated at low and high confluencies for 48 h. **b** N17-Q97P-GFP aggregation in HeLa cells treated as in **a**. Bars represent the mean ± s.d. of one representative experiment performed in triplicates (***P < 0.001, *P < 0.05, NS, not significant; two-tailed *t*-test). The experiment was repeated with similar results. **c** Representative confocal images of N17-Q97P-GFP aggregates in HeLa cells overexpressing control (empty flag vector), YAP(5SA) or YAP(5SA/S94A) were seeded at low and high confluencies and exposed to either control (DMSO) or VPS34-IN1. Scale bars are 10 µm. **d** Quantification of the percentage of HeLa cells with at least one N17-Q97P-GFP aggregates. The cells were treated as in (**c**). Bars represent the mean ± s.d. (*n* = 3; ***P < 0.001, **P < 0.01, *P < 0.05; two-tailed *t*-test). *n* = number of independent biological replicates unless otherwise stated

As reported above, high confluency cells accumulate autophagy substrates, like mutant huntingtin (htt) exon1 (N17-97QP-GFP – Supplementary Fig. 2b). YAP/TAZ downregulation in HeLa cells increased the percentage of cells with htt exon1 N17-97QP-GFP aggregates only in LC conditions; it had no additional effect in HC (Fig. 6a, b). Interestingly, LATS1/2 depletion alone reduced the percentage of cells with mutant htt aggregates in HC cells, but had no effect in cells where YAP/TAZ were knocked down (Fig. 6a, b). We confirmed that the accumulation of mutant htt in HC is YAP/TAZ-dependent, since YAP(5SA) and not YAP(5SA/94 A) reduced the percentage of cells with mutant htt aggregates in HC (Fig. 6c, d). To assess the role of autophagy under these circumstances, we inhibited autophagy with the VPS34 selective inhibitor (VPS34-IN1^53^) and observed that in both YAP(5SA)- and YAP(5SA/S94A)-expressing cells, autophagy inhibition enhanced the percentage of cells with mutant htt aggregates in low confluency cells and abolished the rescue mediated by YAP(5SA) in HC (Fig. 6d). These data suggest that autophagy is downstream of YAP/TAZ in maintaining the appropriate clearance of substrates (like mutant htt) and that the YAP/TAZ-autophagy axis is the main checkpoint in the accumulation of mutant htt in high confluency cells.

### Cells at high density or in soft ECM are prone to apoptosis

**Fig. 7:**
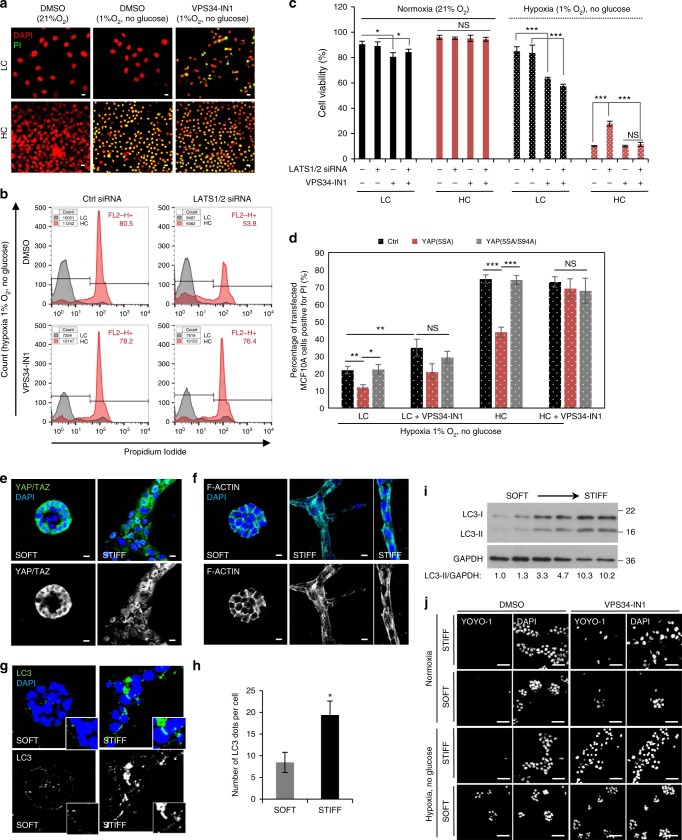
Effects of soft ECM vs. stiff ECM on autophagy regulation. **a** Representative propidium iodide (PI, shown in green) and DAPI (shown in red) staining in MCF10A cells plated at low/ high densities and cultured for the last 24 h in hypoxia and no-glucose conditions, ±VPS34-IN1. The green and red colors were chosen to increase the images’ visibility. **b** Propidium iodide (PI) analysis by flow cytometry in MCF10A cells exposed to LATS1/2 siRNAs and then plated at low/ high densities. For the last 24 h, the cells were grown in hypoxia and no glucose conditions, ±VPS34-IN1 (1 µM). **c** Cell viability analysis using the annexin5-FITC/PI method. MCF10A cells were exposed to LATS1/2 siRNAs and then plated at low/ high densities. For the last 24 h, the cells were grown in either normoxia or hypoxia and no-glucose conditions, ±VPS34-IN1 (1 µM). Bars—means ± s.d. (*n* = 3; *P < 0.05, ***P < 0.001; two-tailed *t*-test). **d** Propidium iodide (PI) staining and cell viability data measured by flow cytometry in high confluency MCF10A cells overexpressing control (empty flag vector), YAP(5SA) or YAP(5SA/S94A). For the last 24 h, the cells were grown in hypoxia and no-glucose,  ±VPS34-IN1 (1 µM). Bars—mean ± s.e.m. (*n* = 4; ***P < 0.001, **P < 0.01, *P < 0.05; two tailed *t*-test). **e** Representative images of YAP/TAZ staining in MCF10A cells plated on either soft or stiff ECM. **f** Representative images of F-actin immunostaining in cells treated as above. **g** Representative images of endogenous LC3 in MCF10A cells seeded on either soft or stiff ECM. **h** Quantification of endogenous LC3 dots in MCF10A cells seeded on either soft or stiff ECM. Bars—mean ± s.e.m. (*n* = 4; *P < 0.05; two-tailed *t*-test), from one representative experiment, where 4 distinct structures of 15–20 cells each where analyzed. **i** Representative LC3-II immmunoblot in MCF10A cells seeded on ECM with different stiffnesses. **j** YOYO-1 staining of MCF10A cells plated on soft or stiff matrixes. For the last 24 h, the cells were grown in hypoxia and no-glucose, ±VPS34-IN1 (1 µM). Scale bars—50 µm. See Supplementary Fig. 18c for quantification of cell apoptosis.Unless otherwise stated, scale bar is 10 µm. *n* = number of independent biological replicates unless otherwise stated

Autophagy assists cell survival during stresses associated with cancerous environments, such as hypoxia and glucose starvation^14,15^. After 24 h in this cancer-mimicking microenvironment, cells plated at high density showed a dramatic increase in the percentage of cells undergoing late stages of apoptosis (propidium iodide-positive) (Fig. 7a, b), as well as a large reduction in the cell viability (expressed as the percentage of cells double-negative for propidium iodide and annexin V)—Fig. 7c. Autophagy inhibition with VPS34-IN1 increased the propidium iodide staining and reduced the cell viability to 60% in low confluency cells subjected to hypoxia and starvation, but had no further effect on high confluent cells in these conditions (Fig. 7c). However, knockdown of LATS1/2 kinases, which induces autophagy by reactivating YAP/TAZ in the HC state (e.g., Fig. 1, and Supplementary Figs. 3, 5, 7, 8) tripled the number of viable high confluency cells, while when combined with the autophagy inhibitor VPS34-IN1, was not able to rescue either the cellular viability or the percentage of cells undergoing apoptosis (Fig. 7c). These data suggest that autophagy inhibition contributes to the cell death resulting from Hippo signaling in high confluency cells in hypoxia and glucose starvation. The fact that knockdown of LATS1/2 kinases only partly rescues the apoptosis phenotype in high confluency cells (Fig. 7b, c), is correlated and likely due to the incomplete rescue of YAP/TAZ nuclear localization and autophagosome numbers (Supplementary Figs. 5d, 8b).

We next investigated these observations using a complementary approach. YAP (S5A) (but not YAP(5SA/S94A)) increased viability of HC HeLa (Supplementary Fig. 15) and MCF10A cells in hypoxia/glucose deprivation conditions (Supplementary Fig. 16). However, when key autophagy genes were downregulated in these cells (Supplementary Figs. 15c, 16c), the rescue effect on cell viability was abolished (Supplementary Figs. 15b, 16b). Similar autophagy-dependent viability effects were seen when we used VPS34-IN1in HC MCF10A cells expressing YAP(5SA) (Fig. 7d and Supplementary Fig. 17a). These hypoxia/glucose deprivation conditions did not inhibit YAP/TAZ function in these two cell lines (Supplementary Fig. 17b–f). These data suggest that the autophagy inhibition resulting from high confluency/YAP-TAZ inactivation contributes to the enhanced sensitivity of such cells to conditions mimicking the tumors microenvironment (hypoxia and glucose deprivation).

The tumor microenvironment promotes collagen deposition in the extracellular matrix (ECM) which increases tissue rigidity (due to high ECM stiffness), favouring tumor growth and tumor cell invasion/metastasis^54^. Stiff ECM activates YAP/TAZ (similar to the 2D low cell confluence state) to promote the growth and invasion of human mammary epithelial cells, causing disorganized acini^8^. However, in an environment that mechanically mimics the normal healthy mammary gland, YAP/TAZ are inactivated due to cytosolic translocation, conferring a phenotype resembling the high confluency state.

We confirmed that YAP/TAZ were preferentially nuclear in stiff ECM with a median apparent elastic modulus *K*_app_ *=* 464 Pa, compared to the cytosolic localization in soft ECM (*K*_app_ = 70 Pa)—Fig. 7e. The actin and autophagy changes mirrored the 2D confluency-dependent phenotype (Fig. 7f–i). Indeed, MCF10A cells embedded in soft ECM resembled high density conditions, showing loss of actin stress fibers (Fig. 7f), reduced numbers of LC3 puncta per cell with decreased LC3-II levels (Fig. 7g–i) and increased p62 levels (Supplementary Fig. 18a, b), when compared to those plated in stiff ECM. Matrix stiffness was assessed using AFM indentation measurements as previously described^55^ –Supplementary Fig. 18b.

As in HC, cells grown in soft ECM showed enhanced apoptosis (the percentage of YOYO1-positive cells) compared to the stiff ECM conditions under hypoxia and glucose starvation (Fig. 7j and Supplementary Fig. 18c–e). However, autophagy inhibition (either chemically—using VPS34-IN1, or genetically—using ATG7/10 or ATG16L1 depletion conditions) of cells grown on stiff ECM under such conditions also increased apoptosis to a similar level as for cells grown on soft ECM (Supplementary Fig. 18c, d). Interestingly, LATS1/2 knockdown was able to reduce the cell death seen in soft matrixes when grown in a glucose- and oxygen-deprivation environment (Supplementary Fig. 18d). These last observations in cells grown on various ECM stiffness (soft vs stiff) resembled the previous findings seen in 2D systems of various cell densities (high vs low) and further strengthen the role of YAP/TAZ regulating cell survival via autophagy under metabolic stress conditions (hypoxia, glucose deprivation).

### Autophagy acts downstream of YAP to control proliferation

**Fig. 8:**
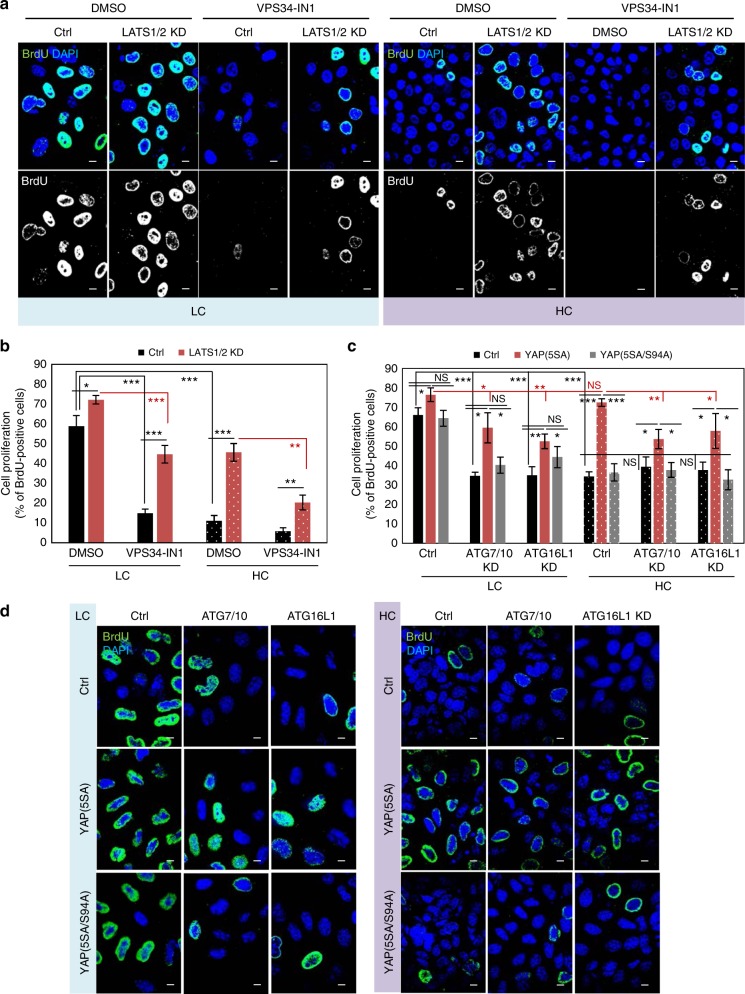
The role of autophagy on YAP/TAZ regulation of cell proliferation. **a** Representative BrdU images of MCF10A cells exposed to LATS1/2 siRNAs, plated at low and high densities and treated with VPS34-IN1 (1 µM) for the last 24 h. Scale bars are 10 µm. **b** Quantification of MCF10A cells positive for BrdU (cell proliferation). MCF10A cells were treated as in **a**. Bars represent the mean ± s.d. (*n* = 3; ***P < 0.001, **P < 0.01; two-tailed *t*-test). **c** Quantification of HeLa cells positive for BrdU (cell proliferation). HeLa cells were initially transfected with control, ATG7/ATG10 or ATG16L1 siRNAs, then transfected with control (empty flag), YAP(5SA) or YAP(5SA/S94A) constructs and plated at low and high confluencies. Bars represent the mean ± s.d. (*n* = 3; ***P < 0.001, **P < 0.01, *P < 0.05, NS not significant; two-tailed *t*-test). **d** Representative BrdU images of HeLa cells overexpressing control (empty flag), YAP(5SA) or YAP(5SA/S94A) for the quantification in **c**. The cells were initially exposed to control, ATG7/ATG10 or ATG16L1 siRNAs. Scale bars are 10 µm. *n* = number of independent biological replicates unless otherwise stated

Next we examined whether autophagy contributed as a downstream effector for the canonical YAP/TAZ functions of modulating cell proliferation. LATS1/2 down-regulation in MCF10A cells increased the percentage of proliferative cells (BrdU positive), an effect that was partially inhibited when the cells were additionally exposed to VPS34-IN1 (Fig. 8a, b) or autophagy depletion conditions such as ATG7/10 or ATG16L1 knockdowns (Supplementary Fig. 19a, b). Interestingly, in low confluency cells, VPS34-IN1 reduced cell proliferation by about 50%, while in cells depleted of LATS1/2 the effect was only 25% (Fig. 8b). Similarly, knockdown of key autophagy genes (either ATG7/ATG10 or ATG16L1) reduced the percentage of BrdU-positive cells by 30% for low confluent HeLa cells expressing either control or YAP(5SA/S94A) and only by 15% in cells expressing YAP(5SA) (Fig. 8c, d). YAP(5SA) overexpression was able to enhance cell proliferation under HC conditions, an effect that was significantly diminished under autophagy inhibition conditions (ATG7/ATG10- or ATG16L1-depletion) (Fig. 8c, d). These results suggest that autophagy promotes proliferation in cells with activated (nuclear) YAP/TAZ.

## Discussion

High cell density or soft ECM promote the contact inhibition of proliferation, the property of cells to cease proliferation and growth when they reach contact with the neighboring cells. The loss of this behavior is a hallmark for malignant transformation, tumor growth, invasion and metastasis. Here we identified that contact-inhibited cells (plated at high cell density) have impaired autophagosome formation. This can be attributed to a drastic reduction in the formation of F-stress fibers and this phenotype is rescued by YAP/TAZ reactivation. The F-actin phenotype is a direct consequence of reduced transcription of the actomyosin genes (MLC2, MYH10, MYH14) caused by YAP/TAZ retention in the cytosol in conditions of hippo signaling activation. While this is likely a major cause of the autophagy phenotype, we cannot exclude additional mediators.

YAP/TAZ activity can be mechanically regulated downstream of actomyosin contractility^56^. Genetically or chemically induced actin depolymerization diminishes tension and thus sequesters YAP/TAZ into the cytosol^7^. Our data argue that YAP/TAZ inhibition by either genetic depletion or mechanical signals at high cell density transcriptionally represses the levels of actomyosin proteins, which results in actin depolymerization, indicating that YAP and TAZ regulate actin dynamics in high confluency cells. We propose a feedforward mechanism that maintains the contact inhibition state (see scheme in Supplementary Fig. 19c): at high cell density, the LATS1/2 kinases are activated and relocalize YAP/TAZ in the cytosol, consequently leading to loss of myosin-II and actin-stress fibers that will further activate LATS1/2 kinases, keeping YAP/TAZ phosphorylated and maintaining their subsequent cytoplasmic retention. This loop will “fix and maintain” the HC-associated hippo signaling state. At low cell density, once the LATS1/2 kinases are inactivated, YAP/TAZ are reactivated and shuttled into the nucleus, rescuing the formation of actin-stress fibers, which will induce relocalisation of YAP/TAZ into the nucleus (independent of LATS1/2) and will maintain the proliferative state. This novel link closes the feedforward loop centered on *actin-YAP/TAZ-actin*. In addition, our results build on this loop and identify that actin depolymerization caused by YAP/TAZ inhibition is responsible for repressed autophagosome biogenesis in the early steps in the process. This actin-autophagy mechanism is supported by previous studies^49,57,58^.

In summary, we have shown that YAP/TAZ modulate autophagy and their inhibition in high confluent cells reduces both proliferation and basal autophagy, thus limiting the general cell metabolism downstream of mTORC1 inhibition. Autophagy inhibition in high confluency cells also leads to the accumulation of long-lived or aggregate-prone proteins in a manner dependent on both YAP/TAZ and autophagy activity. Not surprisingly, these three components: YAP/TAZ activity^5,6,52,59^, actin dynamics^52,60^, and autophagy^14,15^ are all implicated and regulate cancer-related events, such as hypoxia, detachment from the ECM^36,50–52^, invasion and metastasis. Indeed, our data suggest that the inhibition of autophagy by high confluency/soft matrix and the activation of autophagy in cells grown on stiff matrix may regulate key downstream consequences of altered Hippo signaling that are relevant to cancer biology—sensitivity to hypoxia/glucose starvation and proliferation. We observed that both phenomena had a dependency on autophagy in response to varying confluency and associated altered YAP/TAZ signaling. Thus, we reveal autophagy as an effector of key phenotypes in this pathway (see schema in Supplementary Fig. 19c). In this context, our results suggest that cells found in an environment where YAP/TAZ are activated, for example in aggressive breast cancers associated with a stiff extracellular matrix (due to excessive collagen deposition), may have increased autophagy, thus giving them growth, invasion and metastatic advantage.

Clearly, from a practical perspective, our study also highlights that one should be vigilant to changes in cell confluency being a cause of altered autophagy in tissue culture experiments, as this may arise from genetic or chemical perturbations to cell growth or through imprecise seeding.

## Methods

### Antibodies

The following primary antibodies were used: rabbit polyclonal anti-ACTB (A2066, Sigma-Aldrich, WB 1:2000 dilution), mouse monoclonal anti-AKT (no. 2920, Cell Signaling, WB 1:500), rabbit monoclonal anti-phospho-AKT(Ser473) (no. 4060, Cell Signaling, WB 1:500), rabbit polyclonal ATG7 (ab52472, Abcam, WB 1:1000), rabbit polyclonal anti-ATG9A (ab108338, Abcam, IF 1:200), rabbit anti-ATG16L1 (PM040, MBL, WB 1:1000), rabbit monoclonal anti-ATG16L1 (no. 8089, Cell Signaling, IF 1:200), mouse monoclonal anti-BrdU (ab8152, Abcam, IF 1:200), rat monoclonal anti-BrdU (ab6326, Abcam, IF 1:100), goat polyclonal anti-CAPZB (ab1338, Abcam, WB 1:1000), mouse monoclonal anti-CD24 (ab134375, Abcam, IF 1:200), rabbit monoclonal anti-DIAPH1 (no. 5486, Cell Signaling, WB 1:1000), mouse anti-Flag M2 (F3165, Sigma Aldrich, WB: 1:2000), mouse monoclonal anti-GAPDH (ab8245, Abcam, WB 1:5000), rabbit polyclonal anti-LC3 (NB100-2220, Novus Biological, WB 1:3000), mouse monoclonal anti-LC3 (clone 5F10, Nanotools, IF 1:200), mouse monoclonal anti-LAMP1 (clone H4A3, Developmental Studies Hybridoma Bank, IF 1:400), rabbit monoclonal anti-MLC2 (no. 8505, Cell Signaling, WB 1:1000), rabbit monoclonal anti- MYH9 (no. 3403, Cell Signaling, WB 1:1000), rabbit monoclonal anti- MYH10 (no. 3404, Cell Signaling, WB 1:1000), rabbit monoclonal anti- MYH14 (no. 3405, Cell Signaling, WB 1:1000), normal mouse IgG (sc-2025, Santa Cruz Biotechnology, ChIP 5 µg), mouse monoclonal anti-p62 (no. 610832, BD Biosciences, WB 1:300), rabbit monoclonal anti-p70 S6 kinase (no. 9202, Cell Signaling, WB 1:1000), rabbit monoclonal anti-phospho-p70 S6 kinase (no. 9204, Cell Signaling, WB 1:500), mouse monoclonal anti-YAP/TAZ (63.7) (sc-101199, Santa Cruz Biotechnology, IF 1:100), rabbit monoclonal anti-YAP (no. 14074, Cell Signalling, IF 1:200, ChIP 5 µg), rabbit monoclonal anti-phospho-YAP-Ser127 (no. 13008, Cell Signaling, WB 1:500).

The secondary antibodies used for immunofluorescence were conjugated to Alexa Fluor 488, 568, 594 or 647 (Invitrogen). The horseradish peroxidise (HRP)-conjugated secondary antibodies used for western-blotting were: anti-mouse (NA931V, GE Healthcare), anti-rabbit (NA934V, GE Healthcare) and anti-goat (no. 611620, Invitrogen-Life Technologies); the following LICOR secondary antibodies were used: anti-mouse 680 and anti-rabbit 800. Phalloidin-Alexa Fluor 546 and 488 from Invitrogen were used for F-actin staining.

### Plasmids and siRNAs

Pre-designed siRNAs (On-Target plus SMART pool and/or set of deconvoluted oligos—see also Supplementary Table 2) targeting Control (D-001810-10), ATG7 (L-020112-00), ATG10 (L-019426-01), ATG16L1 (L-021033-01), YAP1 (L-012200-00), WWTR1/TAZ (L-012200-00), LATS1 (L-004632-00) and LATS2 (L-003865-00) were purchased from Dharmacon—ThermoScientific. Silencer pre-designed siRNAs targeting human CAPZB were purchased from Ambion (no. 146535, no. 146536 and no. 146537). The following constructs were used in this study: 8XGTIIC-luciferase (no. 34615, Addgene) (34, 47), empty pEGFP, pEGFP-α-synuclein A53T, pEGFP-MLC2 (no. 35680, Addgene), pEGFP-MLC2 (T18D, S19D) (no. 35682, Addgene), MST2-GFP (no. 19056, Addgene).empty pCMV, pCMV-flag YAP2 5SA (no. 27371, Addgene), pCMV-FLAG-YAP-5SA/S94A (no. 33103, Addgene).

### Reagents

Bafilomycin A1 (from Millipore) was resuspended in dimethyl sulphoxide (DMSO from Sigma-Aldrich) and used for blocking autophagosome degradation at either 400 nM for 4–6 h or 200 nM overnight. As bafilomycin A1 (BafA1) is dissolved in DMSO, all conditions without BafA1 received an equivalent volume of DMSO. Blebbistatin (B0560) and verteporfin (SML0534) were purchased from Sigma-Aldrich. VPS34-IN1 (Vps34 Inhibitor, no. 532628) was purchased from Calbiochem. MiR control and miR-375 were purchased from Sigma-Aldrich.

### Cell culture

Human cervical epithelium (HeLa) cells and primary mouse embryonic fibroblasts (MEFs) were grown in DMEM (D6546 Sigma-Aldrich) supplemented with 10% fetal bovine serum (FBS, F7524 Sigma-Aldrich), 100 U ml^−1^ penicillin-streptomycin (P0781 Sigma-Aldrich), 2 mM L-glutamine (G7513 Sigma-Aldrich) at 37 °C and 5% CO_2_, humidified atmosphere. HeLa cells stably expressing either mRFP-GFP-LC3 or GFP-LC3 reporter were grown in the same media supplemented with 600 µg ml^−1^ of G418 (1181-031 Invitrogen). HeLa cells (source ATCC) were authenticated by STR profiling.

Primary mouse mammary epithelial cells (pMECs) were isolated and cultured from C57BL6 mice. Primary cells expressing mRFP-GFP-LC3 (either mouse fibroblasts or mouse mammary epithelial cells) were obtained after crossing the mRFP-GFP-LC3 transgenic mice (created by our laboratory) with C57BL6 mice and pregnant females (E16.5 gestation) were killed and the embyos and/or mammary glands were harvested.

The parental MCF10A cells were purchased from Horizon (Catalogue No. HD PAR-058) and cultured in DMEM:F12 (D6421 Sigma-Aldrich) supplemented with 5% horse serum (H1270 Sigma Aldrich), 0.1 µg ml^−1^ cholera toxin (C8052 Sigma-Aldrich), 20 ng ml^−1^ hEGF (E9644 Sigma-Aldrich), 10 µg ml^−1^ insulin (I9278 Sigma-Aldrich), 0.5 µg ml^−1^ hydrocortisone (H0135 Sigma-Aldrich), 2 mM l-glutamine (G7513 Sigma-Aldrich) and 100 U ml^−1^ penicillin-streptomycin (P0781 Sigma-Aldrich) at 37 °C and 5% CO_2_, humidified atmosphere. 1 mg of Hydrocortisone was resuspended in 1 ml of ethanol, supplemented with 19 ml of media and aliquoted in 5 ml tubes. For cell confluency experiments, 50,000 cells and 10 times more cells per well were plated in a 6 well plate for low and high cell density, respectively. When used 12 well plates, half of the number of cells were plated per well.

HaCaT cells were a gift from Prof. Nick Coleman (University of Cambridge) and were cultured in DMEM (D6546 Sigma-Aldrich) supplemented with 10% fetal bovine serum (FBS, F7524 Sigma-Aldrich), 100 U ml^−1^ penicillin-streptomycin (P0781 Sigma-Aldrich) and 2 mM L-glutamine (G7513 Sigma Aldrich) at 37 °C and 5% CO_2_, humidified atmosphere.

All the cell lines were routinely tested for mycoplasma contamination.

### mRFP-GFP-LC3 mouse fibroblasts

The mRFP-GFP-LC3 transgenic mice were created by Dr F. Menzies under the jurisdiction of appropriate Home Office Project and Personal animal licenses and with local Ethics Committee approval, as previously reported in ref. ^17^.

For primary mRFP-GFP-LC3 mouse fibroblasts, transgenic mice were crossed with C57BL6 mice and pregnant females (E16.5 gestation) were sacrificed and embryos were harvested. The primary mRFP-GFP-LC3 mouse fibroblasts were grown in DMEM (D6546 Sigma-Aldrich) supplemented with 10% fetal bovine serum (FBS, F7524 Sigma-Aldrich), 100 U ml^−1^ penicillin-streptomycin (P0781 Sigma-Aldrich), 2 mM L-glutamine (G7513 Sigma-Aldrich) at 37 °C and 5% CO_2_, humidified atmosphere.

### Primary mouse mammary epithelial cells

All studies and procedures were performed under the jurisdiction of appropriate UK Home Office Project and Personal animal licenses and with the approval of the University of Cambridge Animal Welfare and Ethical Review Body. Primary mouse epithelial cells (pMECs) were isolated and cultured following the protocols described by Matthew J. Smalley^61^ and Chen et al.^62^. At E16.5 gestation, female mice were sacrificed by cervical dislocation and the abdominal and inguinal mammary glands were collected in HBSS. The mammary glands were next transferred in a Petri dish and minced with curved scissors (around 200 cuts). The minced mammary gland were then incubated in 10 ml of DMEM:F12, containing 5% horse serum, 3 mg of Collagenase (C9891 Sigma-Aldrich) and 2.5 mg (around 1000 U) hyaluronidase (H3506 Sigma-Aldrich), on a rotating surface at 37 °C and 5% CO_2_, humidified atmosphere for 2 h. The solution was next collected, transferred in a 15 ml falcon tube and pipetted up-down 10 times to dissociate the MECs from the fat clumps. The tubes were centrifuged at 500 × *g* for 5 min to pellet the cells, while the supernatant with the fat clumps were removed. The cells were resuspended in 10 ml red blood lysis buffer, incubated at room temperature for 5 min and pelleted at 500 *g* (5 min). The red blood lysis step was repeated once more and the sample was transferred in a new 15 ml falcon tube. The samples were washed twice and incubated with DMEM (D6546 Sigma-Aldrich) supplemented with 10% fetal bovine serum (FBS, F7524 Sigma-Aldrich), 100 U ml^−1^ penicillin-streptomycin (P0781 Sigma-Aldrich), 2 mM l-glutamine (G7513 Sigma Aldrich) in a T-75 flask at 37 °C and 5% CO_2_, humidified atmosphere for 1 h, to allow the majority of the fibroblasts to attach to the bottom of the T-75 culture flask. Then, the suspension of epithelial cells was collected in a 15 ml falcon tube, pelleted at 500 *g* for 5 min, washed once with PBS, resuspended and incubated in 3 ml of 0.25% pre-warmed trypsin-EDTA (25200 Invitrogen) at room temperature for 5 min. The trypsin reaction was stopped with 10 ml of DMEM:F12, containing 5% horse serum and 10 g l^-1^ DNase (DN25 Sigma-Aldrich) and the epithelial cells were pelleted at 500 *g*, resuspended and cultured in the MCF10A culture medium: DMEM:F12 (D6421 Sigma-Aldrich) supplemented with 5% horse serum (H1270 Sigma Aldrich), 0.1 µg ml^−1^ cholera toxin (C8052 Sigma-Aldrich), 20 ng ml^-1^ hEGF (E9644 Sigma-Aldrich), 10 µg ml^−1^ insulin (I9278 Sigma-Aldrich), 0.5 µg ml^−1^ hydrocortisone (H0135 Sigma-Aldrich), 2 mM l-glutamine (G7513 Sigma-Aldrich) and 100 U ml^−1^ penicillin-streptomycin (P0781 Sigma-Aldrich) at 37 °C and 5% CO_2_, humidified atmosphere.

### 3D culture of MCF10A cells

The 3D matrix is a mixture of Rat Collagen I (TREVIGEN) and Growth Factor Reduced Matrigel (BD Biosciences), following the procedure described in ref. ^7^. Collagen I was neutralized with 7.5% Sodium Bicarbonate in 1 × PBS (according to the manufacturer’s instructions) on ice and used at 1 mg ml^−1^ (soft matrix) and 3 mg ml^−1^ (stiff matrix) final concentrations. Matrigel was added to the Collagen I solution to constitute 5% of the final volume. The CollagenI-Matrigel solution represented the extracellular matrix (ECM). 1 × volume of ECM was combined with 1 × cold medium and used for coating Nunc 8 chamber slides (150 µl of the mixture was added per well). After the ECM gelled at 37 °C and 5% CO_2_, a mixture of 1 × volume ECM with 1 × volume of cells (2000 cells in 100 µl) was added (in drops) on the top of the pre-gelled ECM (150 µl of final mixture per well). After 2 h (when the ECM-cell mixture gelled), the wells were supplemented with MCF10A culture medium. The wells were replenished every 2 days with culture medium.

### siRNA transfection

For siRNA transfection experiments, HeLa cells were seeded in 6 well plates one day before and transfected with 100 nM of the indicated siRNA using Lipofectamine 2000 (Invitrogen), following the manufacturer’ s protocol. MCF10A cells were transfected with siRNAs using Lipofectamine RNAiMAX (13778150 Invitrogen) instead of Lipofectamine 2000; the rest of the protocol was the same. The following day, cells were again transfected with 50 nM of siRNA. At 48 h post transfection the cells were split and reseeded in 6 well plates according to the experiment’s requirement and cultured in full medium for 48 h. HeLa cells stably expressing the mRFP-GFP-LC3 reporter were transfected following the protocol above, except that at 72 h post transfection the cells were reseeded in 96 or 12 well plates.

### Immunofluorescence microscopy

For immunofluorescence microscopy, cells (HeLa cells or primary mouse cortical neurons) were cultured on coverslips. Cells were fixed in PFA 4% for 5 min, permeabilized with Triton 0.1% for 10 min and then blocked in 3% BSA for 1 h. The cells were washed three times (for 5 min) in PBS and blocked for 1 h in 0.3% BSA (BP1605-100 Fischer Scientific) and incubated with primary antibodies. After conjugation with secondary antibodies tagged with Alexa Fluor for 90 min, the coverslips were briefly rinsed in PBS and mounted in Prolong Gold Antifade reagent (with DAPI; P-36931 Invitrogen). Coverslips were examined with a confocal microscope (63 × NA 1.4 Plan Apochromat oil immersion lens; Carl Zeiss LSM710, LSM780 and LSM880). When necessary, the images were exported in Photoshop (Adobe) and equal adjustments were made for all the images from all control and treatment groups. Each experiment was performed at least twice with similar results.

### BrdU (5-Bromo-2′-deoxyuridine) staining

Cells were plated and grown on coverslips. Freshly prepared BrdU was diluted to 10 µM in pre-warmed culture media and added to the cells for 2 h. Then, the media was removed and the cells fixed in 4% PFA for 30 min at room temperature. After fixation, the cells were washed 3 times with PBS (5 min each wash) and exposed to 1.5 M HCl for 1 h. The cells were next rinsed several times with PBS and incubated in the blocking buffer (5% goat serum and 0.3% TritionX-100 in 1 × PBS) for 90 min. The primary antibody was added at 1:200 dilution, in 5% goat serum in PBS, at 4 °C overnight. The following day, the cells were washed three times with PBS, incubated with the secondary antibody (at 1:400 dilution) and mounted in the Prolong Gold Antifade reagent. More than 600 cells were counted per condition, per experiment.

### Automated fluorescence microscopy

Automated fluorescent analysis was performed using a Cellomics microscope (Thermo-Fisher Scientific). The images were analyzed using the Spot Detector module in order to quantify the number and size of autophagic vesicles (either GFP-LC3 or mRFP-LC3). The acquisition thresholds were set up such that the background signal was removed. All the acquisition settings were kept unchanged between various conditions (controls and treatments) analyzed. More than 600 cells were counted per condition, per experiment.

### AFM indentation measurements

AFM measurements were carried out similarly as previously described^55^. Monodisperse polystyrene beads (radius *r* = 18.64 µm ± 0.17 µm, microParticles GmbH, Berlin, Germany) were glued to tipless silicon cantilevers (spring constants between 0.01 and 0.03 N m^−1^; Arrow-TL1, NanoWorld, Neuchatel, Switzerland). The AFM was mounted on an *x*/*y* motorized stage of an inverted microscope (AxioObserver A1, Zeiss, Cambridge, UK). Phase-contrast microscopy was used simultaneously to visualize the position of the substrate. Force-distance curves were taken with a force of 10 nN at room temperature (>20 curves for each collagen gel, 4 different gels per stiffness). The apparent elastic modulus (*K*_app_) was calculated using the Hertz model: F = 4/3 ***K r***^**1**/**2**^ ***δ***^**3**/**2**^ for an indentation depth µ = 2 µm, using a custom written automated algorithm based in Matlab (MathWorks, Natick, USA). Statistical significance was determined using a Kruskal-Wallis test.

### Cell lysis and western blot analysis

Cells were seeded and cultured in 6 or 12 well plates. Cells were washed twice in cold phosphate buffered saline (PBS) and either lysed in RIPA buffer containing 150 nM NaCl, 1% NP40, 0.5% NaDoc, 0.1% SDS, 50 mM Tris, (all from Sigma Aldrich), protease inhibitors mix and phosphatase inhibitors from Roche Diagnostics or directly in 2 × Laemmli Sample Buffer (161-0737 BioRad). The samples lysed in RIPA buffer were incubated for 30 min on ice to ensure complete lysis and then centrifuged at 16,200 × *g* for 15 min at 4 °C to pellet the debris. The supernatants were collected in separate tubes and the protein levels were quantified using BioRad Protein Assay Kit II (BioRad)—an assay based on the Bradford method, and normalized for loading. Cell lysates were further diluted in 2 × Laemmli Sample Buffer (161-0737 BioRad) and boiled for 7–10 min at 100 °C. For western-blot analysis, the samples were subjected to an SDS-PAGE separation (80 V for the time the samples are running in stuck gel and 110 V for the rest of the time), followed by transfer on PDVF membranes using the BioRad mini gel system (95 V for 60–90 min, depending on the protein of interest). The membranes were blocked in 6% milk in 0.1% Tween in PBS for 1 h and incubated with primary antibody at 4 °C overnight. Incubation with secondary antibody for 90 min was followed by protein visualization either with the ECL detection kit (GE Healthcare) or directly on a LI-COR Odyssey scanner. Proteins levels detected by ECL were quantified using ImageJ software, while proteins detected by IRdye were quantified using Image Studio Software (LI-COR). Full blot images are shown for important blots in Supplementary Fig. 20.

### Quantification of polyQ aggregation

N17-97QP-GFP aggregation was monitored with a fluorescence microscope. The counting method has been previously described^17^. More than 300 cells were counted per coverslip and the proportion of cells with at least one aggregate was scored out of total number of transfected cells. The experiments were performed in triplicates without knowing the identity of the slides.

### α-synuclein A53T assay

MCF10A and HeLa cells were transfected with 1.5 μg of pEGFP-α-synuclein A53T and 0.5 μg of empty pEGFP per well in a 6 well plate, as previously described^24^. After 12 h of transfection, the cells were seeded at low and high confluencies for 48 h. Then, cells were lysed and analyzed by western-blotting for GFP levels of both GFP-α-synuclein A53T and empty GFP; the ratio between these two signals was calculated.

### Luciferase reporter assay

The luciferase reporter construct 8xGTIIC-LUC was purchased from Addgene (#34615)^8^. MCF10A or HeLa cells were transfected with 0.4 µg of luciferase reporter together with 0.04 µg of renilla luciferase and cultured in full medium for 24 h. The cells were lysed in the reporter lysis buffer provided by Promega and the firefly and renilla luciferase activities were measured using the Dual-Glo luciferase assay system kit (Promega E1910) according to the manufacturer’s protocol. The relative luciferase activity is computed as a ratio between the firefly and renilla luminescence values as described in Wu et al.^63^.

### RNA isolation and quantitative real time PCR

Total RNA was isolated from HeLa cells using TRIzol reagent (15596018 Invitrogen). The RNA sample was then treated with DNase I Amplification Grade (18068-015 Invitrogen) to remove the contaminant DNA. The prepared RNA was reversed transcribed in cDNA with SuperScript III First Strand Synthesis System for RT-PCR (1880-051 Invitrogen) following the manufacter’s instructions. The synthesized cDNA was further diluted 1:10 in DNase Free water, mixed with SYBR Green PCR Master Mix (4309155 Applied Biosystems) and processed by real time qPCR using a 7900 Fast Real-time PCR System (Applied Biosystems). The mRNA quantification of the target gene was based on the ΔΔCT method and the expression levels were plotted relative to GAPDH mRNA. The primer sequences were purchased from Invitrogen and listed in Supplementary Table 3.

### Chromatin immunoprecipitation

5 × 10^7^ MCF10A cells, previously plated at either low or high cell densities, were cross-linked in 1% formaldehyde (F8775 Sigma-Aldrich) for 10 min, exposed to 0.2 M Glycine for 5 min at 25 °C (to stop the cross-linking) and washed twice with PBS. Cells were lysed in 1 ml of Buffer I (10 mM Tris pH 8.0, 10 mM NaCl, 0.2% NP40), containing protease inhibitors and 10 mM NaBu, on ice for 10 min. The nuclei were pelleted by centrifugation at 600 × *g* for 5 min at 4 °C, resuspended in 1 ml Buffer II (50 mM Tris pH 8.0, 10 mM EDTA, 1% SDS), containing protease inhibitors and 10 mM NaBu and incubated for 10 min on ice. The nuclei solutions were transferred in a 10 ml tube and diluted 1:1 in Buffer III (20 mM Tris pH 8.0, 2 mM EDTA, 150 mM NaCl, 1% Triton X-100, 0.01% SDS). The samples were next sonicated for 10 cycles of 30 s on with 30 s off, transferred in a new tube and 3 ml of Buffer III were added to dilute the sheared chromatin. The tubes were next centrifuged at 1500 × *g* for 10 min at 4 °C, to clean up the supernatant containing the chromatin. Next, the chromatin was precleared with IgG (2 µg ml^−1^) for 1 h. 900 µl out of the 5 ml of sheared chromatin was incubated with either YAP1 antibody or IgG overnight at 4 °C on a rotating wheel, while 10% (equivalent to 90 µl) were kept as input. The following day, the immune-complexes were isolated with Protein G sepharose beads (17-0618-01 GE Healthcare) and washed twice with washing buffer A (20 mM Tris pH 8.0, 2 mM EDTA, 50 mM NaCl, 1% Triton X-100, 0.1% SDS), twice with washing buffer B (10 mM Tris pH 8.0, 1 mM EDTA, 0.25 M LiCl, 1% NP40, 0.1% Sodium Deoxycholate) and once with TE buffer. The DNA–protein–antibody complexes were eluted from the sepharose beads with 300 µl of Buffer IV at 25 °C. The cross-linking was reversed by treated the sample with RNase A (1 µl for each sample of 300 µl) and NaCl (0.3 M) at 65 °C overnight. The next day, the DNA was purified using the QIAquick Purification Kit (28704 Qiagen) and used for qPCR quantification and analysis. See also schema in Supplementary Fig. 5l. The samples were normalized to input values (obtained from standard curves).

The primer sequences used for ChIP-qPCR were: MLC2 (Fw: 5′-CTGGACATTCCGTTTGCCTC-3′; Rv: 5′-CCCCCTTCCTTGCTGTGC-3’), MYH10 (Fw: 5′-GAGCCTATGACGGGCGTAAG-3′; Rv: 5′-GTTAGCAACCCGAACGAAGC-3’), MYH14 (Fw: 5′- TTCTACCTTTGTCACCGGGC-3′; Rv: 5′-TGGTTTGCCTCGGGTTCTTT-3′), ACTB (Fw: 5′-CTCTAGGCTGAGCCGAATGC-3′; Rv: 5′-GAGTAGCACACAAGACCGGG-3′), CYR61 (Fw: 5’-AGCAAACAGCTCACTGCCTT-3′; Rv: 5′-ATGGTAGTTGGAGGGTCGTG-3′).

### Lysosomal pH assay

LysoSensor Yellow/Blue DND-160 (L-7545 Molecular Probes) probe was added at 1.5 μM in full medium for 20 min, as previously described^17^. Cells were visualized with a confocal microscope and the fluorescence intensities in the blue and yellow channels were quantified in ImageJ.

### Apoptosis detection assays

MCF10A and HeLa cells were analyzed for apoptosis using the (Annexin V-FITC/PI Kit, Abcam) as previously described^24^. Briefly, cells were plated at low or high densities and cultured for 48 h before glucose starvation and hypoxia (1% O_2_) for 24 h. In total 2 × 10^5^ cells were gently trypsinized, collected by centrifugation (500 × *g*, 3 min), resuspended in 500 µl of binding buffer and incubated with 5 µl annexin V (FITC) and 5 µl propidium iodide (PI) for 5 min. The annexinV-FITC/PI staining was analyzed by flow cytometry using appropriate compensation settings. For all experiments, HeLa and MCF10A cells were transfected with constructs and/or siRNAs before being split at low and high confluencies.

To quantify cell viability in MCF10A cells seeded in soft and stiff matrices, YOYO-1 (Y3601, Life Technologies) was used following the manufactor’s instructions and the coverslips were imaged by confocal microscopy.

### Bioinformatics analysis

For identifying novel potential YAP/TAZ targets, we combined chromatin-immunoprecipitation data from 3 sources^6,64,65^ and found 5326 genes whose promoter regions were enriched in both YAP/TAZ and TEAD transcription factors. This data was analyzed for gene ontologies by using Panther and Cytoscape-BINGO. The TEAD binding motifs were searched in the promoter of the mentioned genes (MLC2, MYH10, MYH14, ACTB) −1500 bp ± 200 bp from the TSS.

### Statistical analysis

Densitometry of western blot bands was performed using either Image Studio (LI-COR) or ImageJ (for ECL-protein revealing). When using the Image J software, each image was set to 8 bits format, inverted and the background was subtracted before measuring the band intensity in ImageJ. The graphs show the mean from independent experiments unless otherwise specified (SD is standard deviation and SEM is standard error of the mean). Unless otherwise stated, *n* equals the number of independent biological replicates. Each experiment was performed at least twice. To quantify the significance of different levels between two groups (control and one treatment condition), the *P*-values were determined either with one sample *t*-test, where the control was initially set up to 100% (when the data from multiple experiments were combined) or two-tailed/one-tailed Student’s *t*-test in Excel software, otherwise. Two-way ANOVA was used to compute the significance levels when multiple conditions were compared.

### Data availability

Authors can confirm that all relevant data are included in the paper and/or its Supplementary Information files.

## Electronic supplementary material

Supplementary Information
