# Supplementary material for: Contact inhibition controls cell survival and proliferation via YAP/TAZ-autophagy axis
Source: Nat Commun. 2018 Jul 27;9:2961. doi: 10.1038/s41467-018-05388-x (PMC6063886; doi:10.1038/s41467-018-05388-x)
Supplement: Supplementary file 1 — Supplementary Information [file 41467_2018_5388_MOESM1_ESM.pdf]

Supplementary Information

Contact inhibition controls cell survival and proliferation via YAP/TAZ-autophagy axis

Pavel et al.

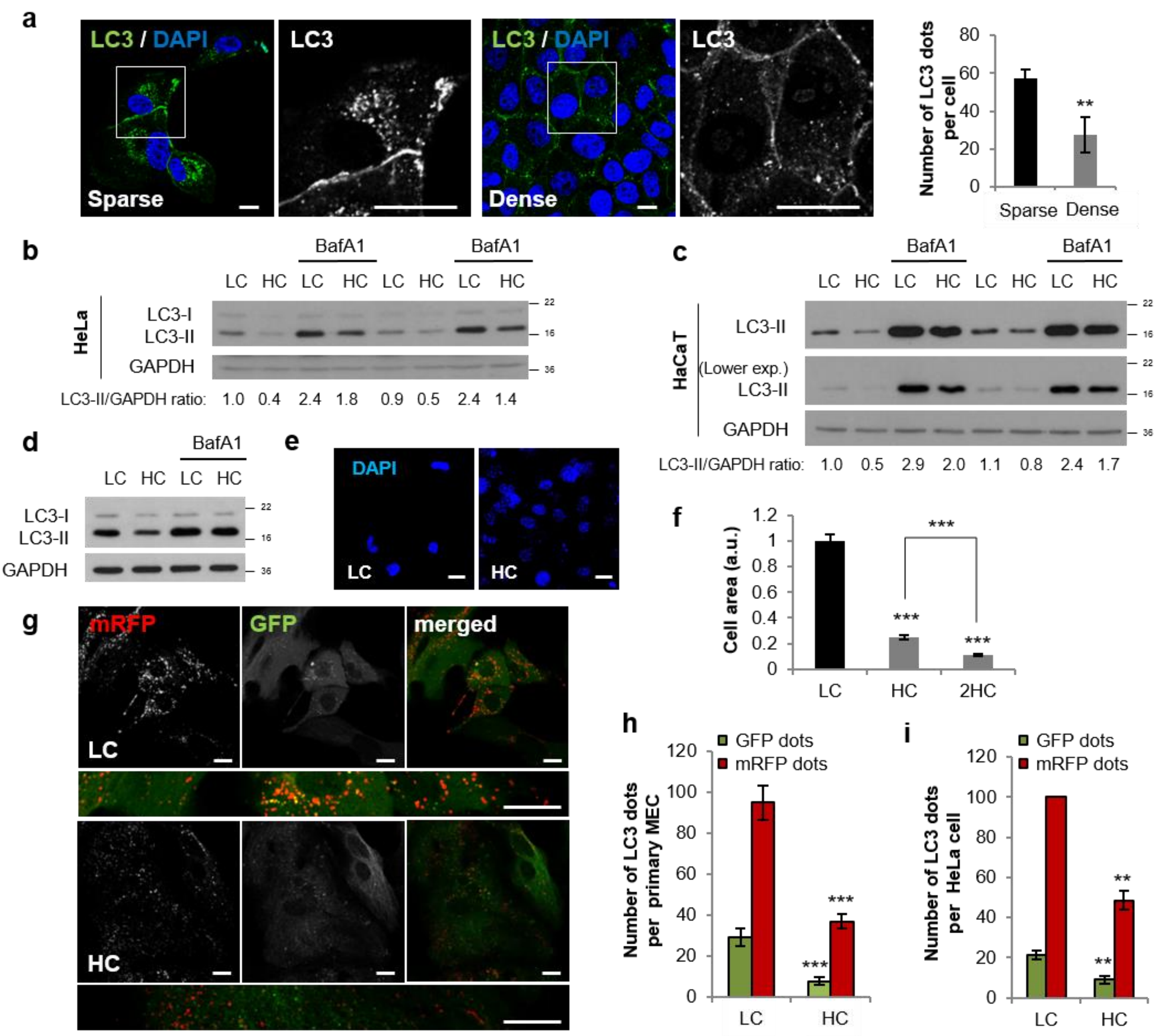

Supplementary Fig. 1: High confluency cells have fewer autophagosomes and accumulate aggregate-prone proteins/ autophagy substrates when compared to low confluency cells.

(a) Endogenous LC3 immunostaining in MCF10A cells plated on the same coverslip. Areas with sparse and dense cells were imaged by confocal microscopy. Quantification of LC3 dots per cell (more than 30 cells were counter per condition) is shown in the right panel (***P*<0.01; two-tailed t-test).

(b) Representative LC3-II immunoblotting at low and high confluencies in HeLa cells. HeLa cells plated at various densities were cultured for 48 hours and exposed to either DMSO or BafA1 (400 nm) for the last 6 hours.

(c) Representative LC3-II immunoblotting at low and high confluencies in HaCaT cells. HaCat cells plated at various densities were cultured for 48 hours and exposed to either DMSO or BafA1 (400 nm) for the last 6 hours.

(d) Representative LC3-II immunoblotting at low and high confluencies in primary MEFs. Primary MEFs plated at various densities were cultured for 48 hours and exposed to either DMSO or BafA1 (400 nm) for the last 6 hours.

(e) DAPI staining for the experiment described in **(d)**.

(f) Average cell area of MCF10A cells plated at LC, HC and 2HC. At least 20-30 cells were quantified per condition. Bars represent the mean±s.e.m. (****P*<0.001; two-tailed t-test).

(g) Representative confocal images of primary mammary epithelial cells (pMECs) from transgenic mice expressing mRFP-GFP-LC3 plated at different confluencies: LC and HC. Scale bar is 10 µm.

(h) Quantification of total number of GFP (autophagosomes) and mRFP dots (autophagosomes and autolysosomes) in pMECs treated as in (**h**). At least 20-30 cells were quantified per condition. Bars represent the mean±s.e.m. (***P<0.001; two-tailed t-test).

(i) Number of GFP (autophagosomes) and mRFP dots (autophagosomes and autolysosomes) in HeLa cells. HeLa cells stably expressing mRFP-GFP-LC3 were seeded at LC and HC and subjected to automated Cellomics visualization and analysis. Bars represent the mean±s.d. (n=3; **P<0.01; two-tailed one sample t-test). More than 600 cells were counted per condition, per experiment.

Note: Throughout the figures, n = number of independent biological replicates unless otherwise stated.

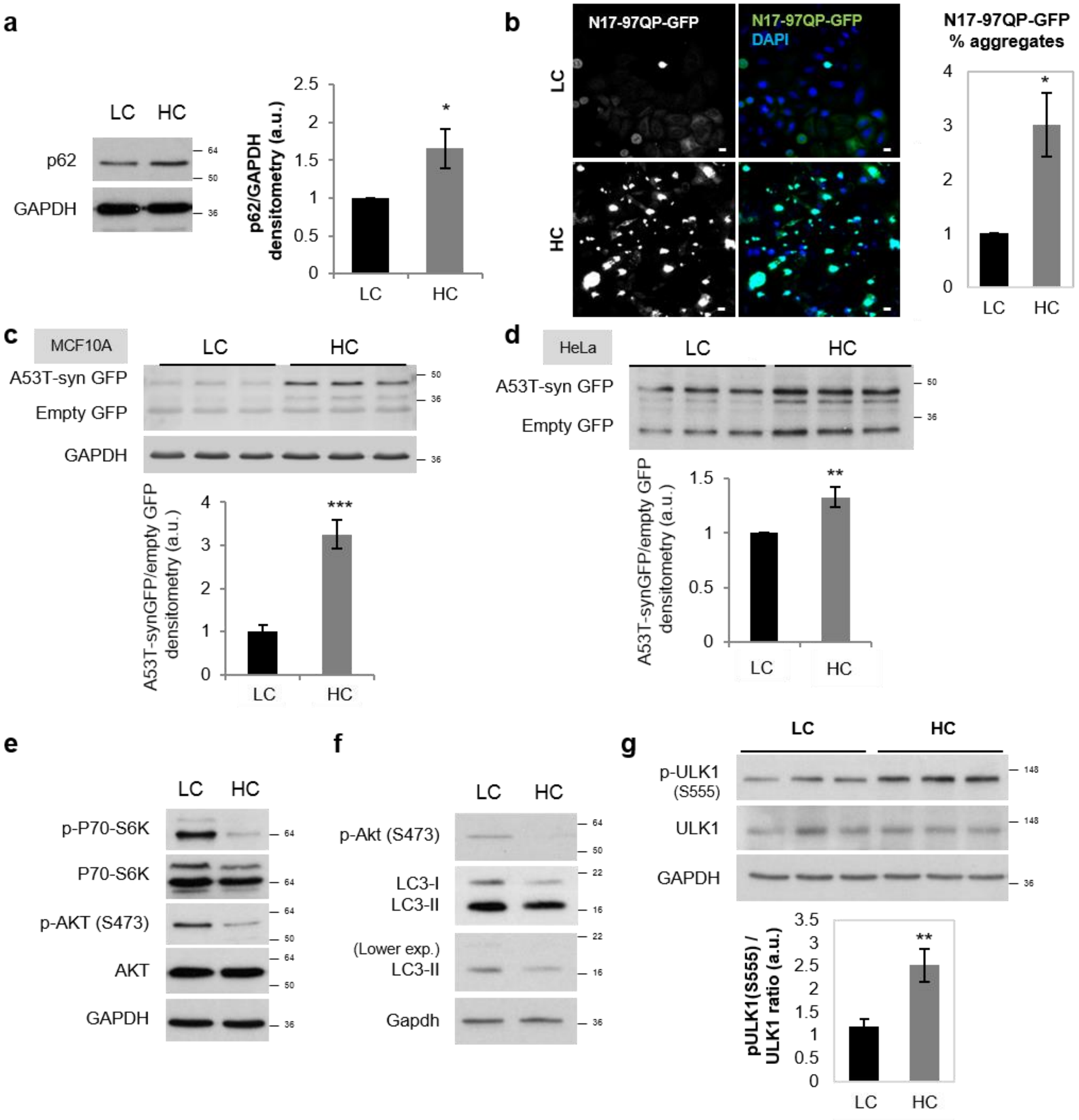

Supplementary Figure 2: High confluency cells have increased levels of aggregate-prone proteins/ autophagy substrates when compared to low confluency cells, despite mTORC1 inhibition.

(a) Representative western-blot of p62 in MCF10A cells. GAPDH was used as loading control.

Quantification of p62 densitometry relative to GAPDH is shown on the right. Bars represent the mean±s.d. (*n*=3; **P*<0.05; two-tailed one sample t-test).

(b) Representative confocal images of HeLa cells transfected with N17-97QP-GFP and then reseeded at low (LC) and high (HC) confluencies. Bars represent the relative ratio of percentages of cells with aggregates between HC and LC conditions (*n*=3; **P*<0.05; two-tailed t-test). Scale bar is 10 μm.

(c) Representative western-blot of A53T-synuclein tagged with GFP in MCF10A cells. GAPDH was used as loading control. Quantification of A53T-synGFP densitometry relative to empty GFP is shown on the bottom panel:

mean±s.d. (one representative experiment in triplicates: ****P*<0.001; two-tailed t-test). The experiment was repeated with similar results.

(d) Representative western-blot of A53T-synuclein tagged with GFP in HeLa cells. Quantification of A53T-synGFP densitometry relative to empty GFP is shown on the bottom panel: mean±s.d. (one representative experiment in triplicates: ***P*<0.01; two-tailed t-test). The experiment was repeated with similar results.

(e) Representative western-blot of the indicated mTORC1 (P70-S6K) and mTORC2 (AKT) substrates in MCF10A cells. GAPDH was used as loading control.

(f) Representative western-blot of the mTORC2 substrate, pAkt(S473) in primary MEFs. Gapdh was used as loading control.

(g) Representative western-blot of pULK1(S555) (a well-characterised AMPK substrate) in MCF10A cells. Quantification of pULK1(S555) densitometry relative total ULK1 levels is shown on the bottom panel: mean±s.d. (one representative experiment in triplicates: ***P*<0.01; two-tailed t-test). The experiment was repeated with similar results.

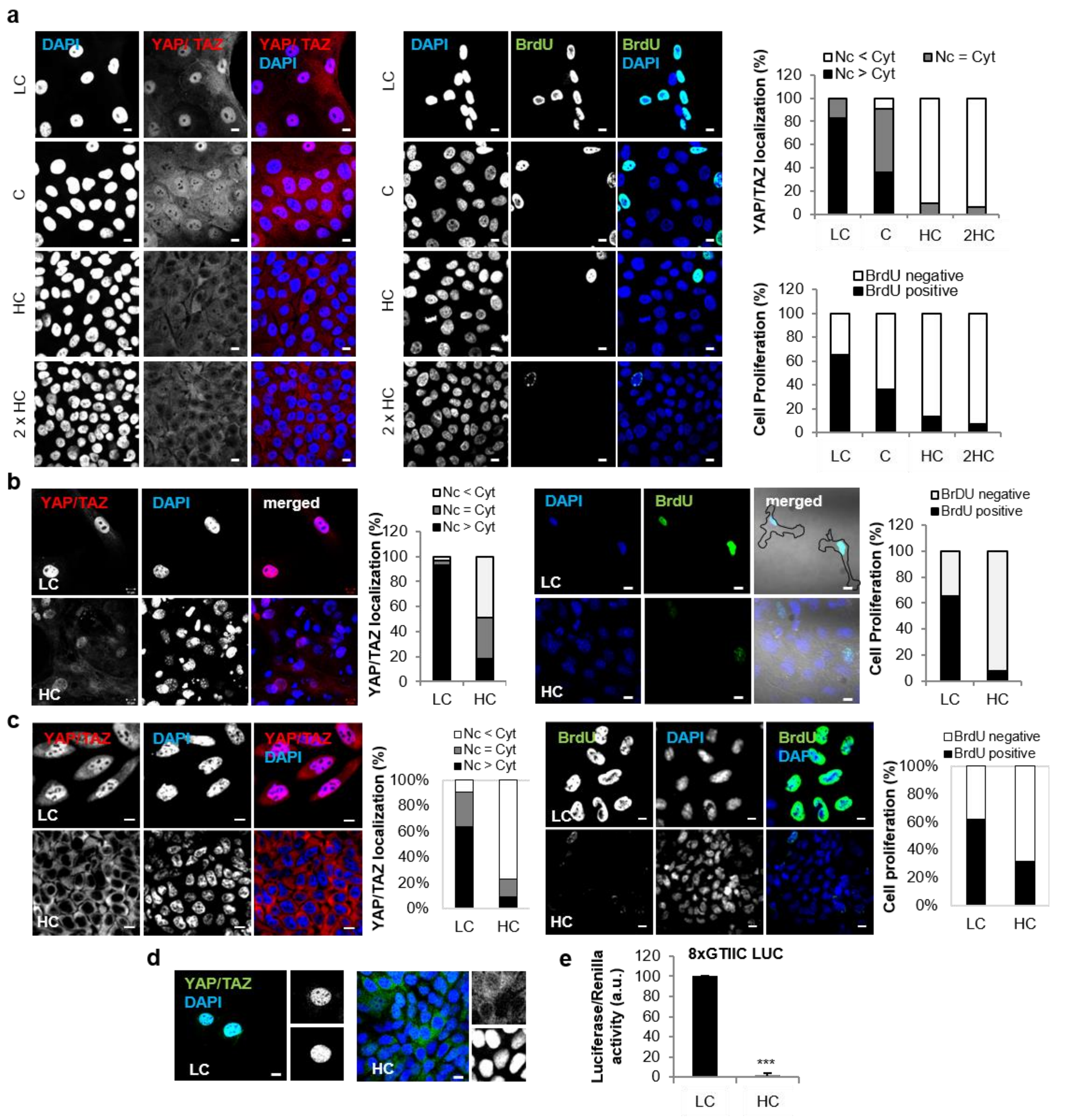

Supplementary Fig. 3: YAP/TAZ localization and proliferation at different cell confluencies.

(a) Representative images of YAP/TAZ and BrdU immunostainings in MCF10A cells seeded at increasing confluencies. Percentages of cells with either nuclear (Nc) or cytoplasmic (Cyt) localization for YAP/TAZ are shown on the top graph. Percentages of cells with positive BrdU immunostaining are shown on the bottom panel. Scale bar throughout the panel is 10 µm.

(b) Representative images of YAP/TAZ and BrdU immunostainings in primary MEFs seeded at low and high confluencies. The graphs show the percentages of cells with nuclear or cytoplasmic localization and the percentages of BrdU-positive cells. Nc = nucleus, Cyt = cytosol. Scale bar is 10 µm.

(c) Representative images of YAP/TAZ and immunostaining in HeLa cells seeded at low and high confluencies. The graphs show the percentages of cells with nuclear or cytoplasmic localization (more than 400 cells were counted per condition) and the percentages of BrdU-positive cells (more than 600 cells were counted per condition). Nc = nucleus, Cyt = cytosol.

(d) Representative images of YAP/TAZ immunostaining in HaCaT cells seeded at low and high confluencies.

(e) Luciferase assay for YAP/TAZ activity in MCF10A cells plated at low (LC) or high (HC) confluency as described in Methods.

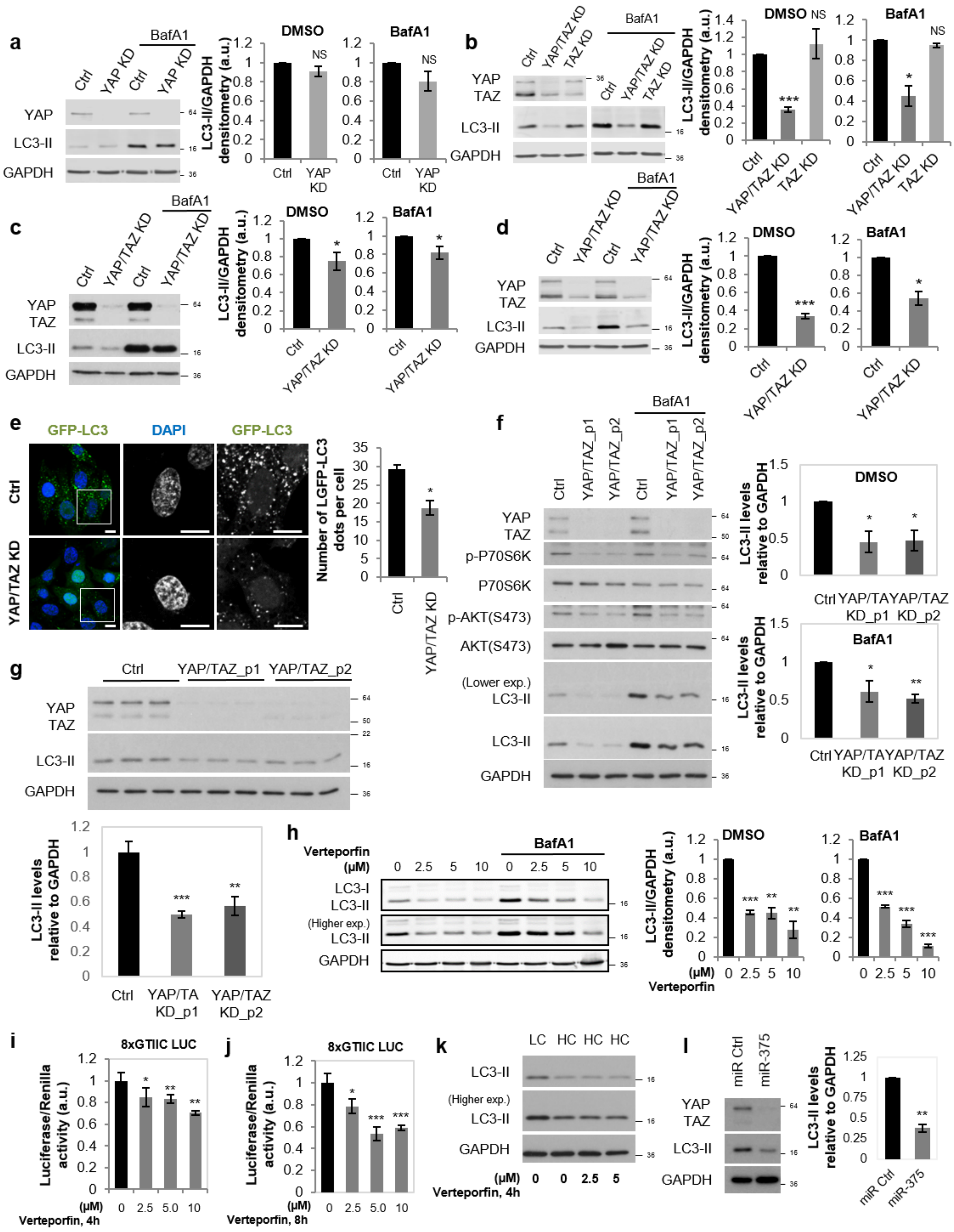
a
b
c
d
e
f
g
h
i
j
k
l
BafA1
DMSO
YAP
TAZ
LC3-II
GAPDH
YAP/TAZ KD
GFP-LC3
DAPI
Number of LGFP-LC3 dots per cell
LC3-II/GAPDH densitometry (a.u.)
LC3-II levels relative to GAPDH
p-P70S6K
P70S6K
p-AKT(S473)
AKT(S473)
Verteporfin (μM)
8xGTIIC LUC
Luciferase/Renilla activity (a.u.)
Verteporfin, 4h
Verteporfin, 8h
miR Ctrl
miR-375

Supplementary Fig. 4: YAP and TAZ double knockdown compromises autophagy.

(a) LC3-II levels assessed by immunoblotting in MCF10A cells exposed to either control (Ctrl) or YAP siRNAs. The cells were treated with vehicle (DMSO) or bafilomycin A1 (BafA1). GAPDH was used as loading control. The graphs show the LC3-II/GAPDH densitometry: mean±s.e.m. (*n*=5; NS - not significant; two-tailed one sample t-test).

(b) LC3-II levels assessed by immunoblotting in MCF10A cells exposed to control (Ctrl), YAP/TAZ (both genes knocked down) or TAZ siRNAs. The cells were treated with vehicle (DMSO) or bafilomycin A1 (BafA1). GAPDH was used as loading control. The graphs show the LC3-II/GAPDH densitometry: mean±s.d. of one representative experiment (*n*=3; ***P<0.001, *P<0.05, NS - not significant; two-tailed one sample t-test).

(c) LC3-II levels assessed by immunoblotting in MCF10A cells exposed to either control (Ctrl) or YAP/TAZ siRNAs (YAP and TAZ double knockdown). The cells were treated with vehicle (DMSO) or bafilomycin A1 (BafA1) at 200 nM for 12 hours. GAPDH was used as loading control. The graphs show the LC3-II/GAPDH densitometry: mean±s.d. (*n*=3 independent experiments performed in triplicates; *P<0.05; two-tailed one sample t-test).

(d) LC3-II levels assessed by immunoblotting in HeLa cells exposed to either control (Ctrl) or YAP/TAZ siRNAs. The cells were treated with vehicle (DMSO) or bafilomycin A1 (BafA1) at 200 nM for 12 hours. GAPDH was used as loading control. The graphs show the LC3-II/GAPDH densitometry: mean±s.e.m. (*n*=4; ***P<0.001, *P<0.05; two-tailed one sample t-test).

(e) Representative images of GFP-LC3 dots in HeLa cells stably expressing GFP-LC3. HeLa cells were exposed to either Ctrl or YAP/TAZ siRNAs. Quantification of GFP-LC3 dots numbers is shown on the right panel (*n*=6 wells of at least 100 cells counted per well; *P<0.05; two-tailed t-test). Scale bar throughout the panel is 10 µm.

(f) Representative LC3-II immunoblots (longer and shorter exposures) assessed in HeLa cells exposed to either control (Ctrl) or individual YAP + TAZ siRNA oligos for 96 hours (pair 1 (YAP/TAZ_p1) and pair 2 (YAP/TAZ_p2), respectively). The cells were treated with vehicle (DMSO) or bafilomycin A1 (BafA1). GAPDH was used as loading control. The graphs show the LC3-II/GAPDH densitometry: mean±s.e.m. (*n*=3; ***P<0.001, **P<0.01; two-tailed one-sample t-test).

(g) Representative LC3-II immunoblot assessed in HeLa cells exposed to either control (Ctrl) or individual YAP and TAZ siRNA oligos for 36 hours (pair 1 (YAP/TAZ_p1) and pair 2 (YAP/TAZ_p2), respectively). GAPDH was used as loading control. Bars represent the mean±s.d. (one representative experiment in triplicates: ***P<0.001, **P<0.01; two-tailed t-test).

(h) Representative LC3-II immunoblot in HeLa cells exposed to increasing concentrations of verteporfin for 16 hours. LC3-II levels were quantified in the absence or presence of bafilomycin A1 (400 nM) for the last 4 hours. The experiment was performed in triplicates and repeated at least twice with similar results: mean±s.d. (***P<0.001, **P<0.01; two-tailed one sample t-test).

(i) Luciferase assay for YAP/TAZ activity in HeLa cells exposed to increasing concentrations of verteporfin for 4 hours. Bars represent the mean±s.d. (**P<0.01, *P<0.05; one-way ANOVA).

(j) Luciferase assay for YAP/TAZ activity in HeLa cells exposed to increasing concentrations of verteporfin for 8 hours. Bars represent the mean±s.d. (***P<0.001, *P<0.05; one-way ANOVA) .

(k) Representative LC3-II immunoblot assessed in high confluency cells exposed to increasing concentration of verteporfin (0, 2.5, 5 µM) in the presence of BafA1.

(l) Representative LC3-II immunoblot assessed in MCF10A cells transfected with either miR control or miR-375. Bars represent the mean±s.e.m. (*n*=4; **P<0.01; two-tailed one-sample t-test).

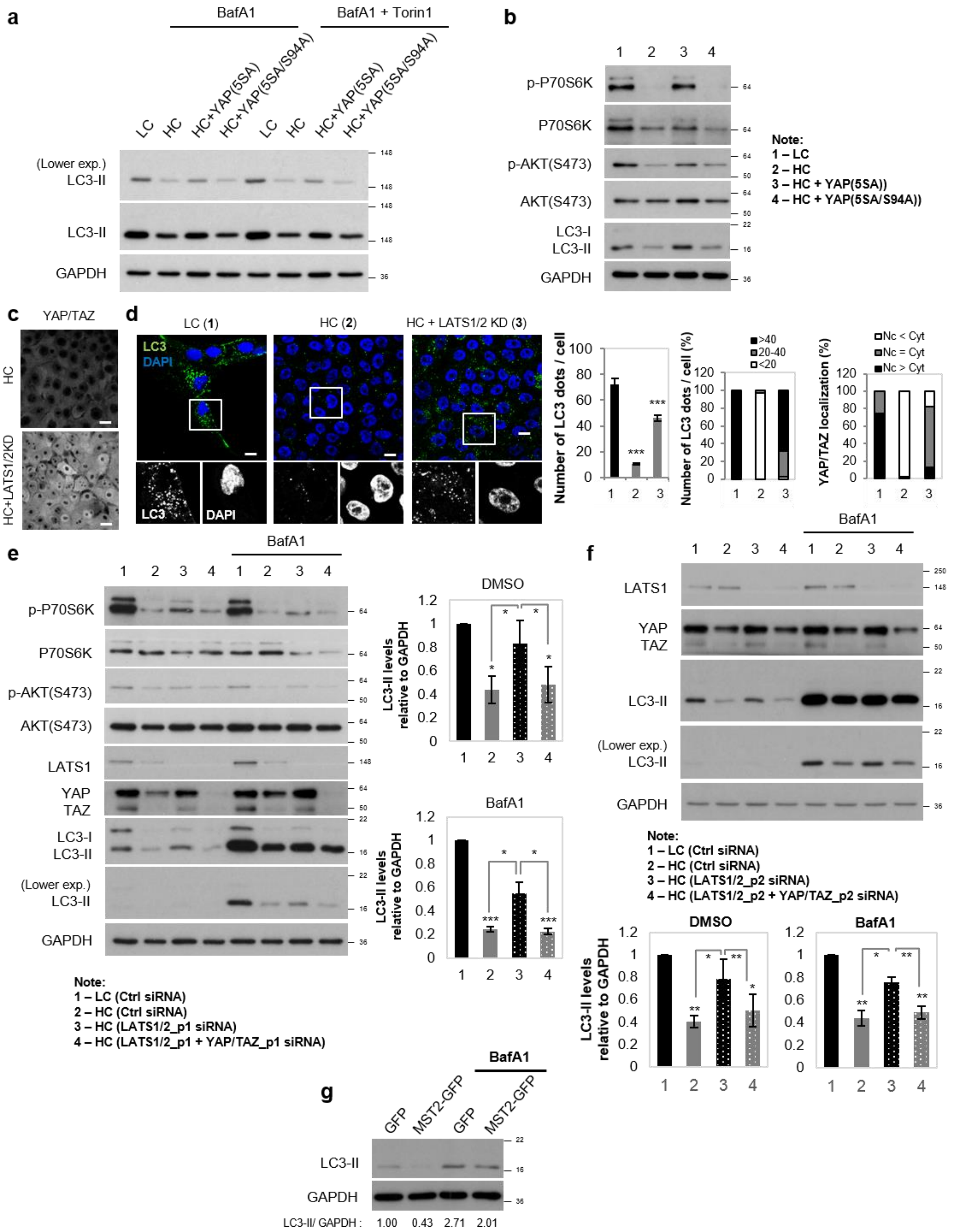

Supplementary Fig. 5: LATS1/2 knockdown rescues the autophagy defects seen in high confluency cells *via* YAP/TAZ activation.

(e) Representative LC3-II immunoblot in HeLa cells overexpressing either YAP(5SA) or YAP(5SA/S94A) in the presence of BafA1. Cells were either or not exposed to Torin1 (used as mTORC1 inhibitor). See **Fig. 1h** for LC3-II quantification.

(b) Representative western-blot of the indicated mTORC1 and mTORC2 substrates in HeLa cells overexpressing either YAP(5SA) or YAP(5SA/S94A).

(c) Representative images of YAP/TAZ immunostaining in highly confluent MCF10A cells exposed to LATS1/2 double knockdown. Scale bar is 10 µm.

(d) Representative images of endogenous immunostaining of LC3 in MCF10A cells treated as in **(c).** Quantification of number of LC3 dots per cell is shown in the bottom panel, presented either as the mean±s.d. (***P<0.001; two-tailed one sample t-test) or as percentages of cells with more than 20 dots per cell. More than 35 cells were counted per LC condition and more than 70 cells were counted per HC condition. The experiment was repeated with similar results at least another two times. YAP /TAZ cytoplasmic localization at high cell density is rescued by LATS1/2 KD (Nc = nucleus; Cyt = cytosol). See Fig. 1k.

(e) Representative immunoblots of the indicated proteins and LC3-II/GAPDH densitometry in MCF10A cells exposed to either control or individual pairs of LATS1 + LATS2 (LATS1/2_p1) and YAP + TAZ (Y/T_p1) siRNA oligos. The cells were treated with vehicle (DMSO) or bafilomycin A1 (BafA1). Bars represent the mean±s.d. (n=3: ***P<0.001, *P<0.05; two-tailed one sample t-test). Distinct oligos were used in parallel experiment in **(f)**.

(f) Representative immunoblots of the indicated proteis and LC3-II/GAPDH densitometry in MCF10A cells exposed to either control or individual pairs of LATS1 + LATS2 (LATS1/2_p2) and YAP + TAZ (Y/T_p2) siRNA oligos. The cells were treated with vehicle (DMSO) or bafilomycin A1 (BafA1). Bars represent the mean±s.d. (n=3; **P<0.01, *P<0.05; two-tailed one sample t-test). Distinct oligos were used in parallel experiment in **(e)**.

(g) LC3-II in cells overexpressing MST2-GFP.

For all immunoblots, GAPDH was used as loading control.

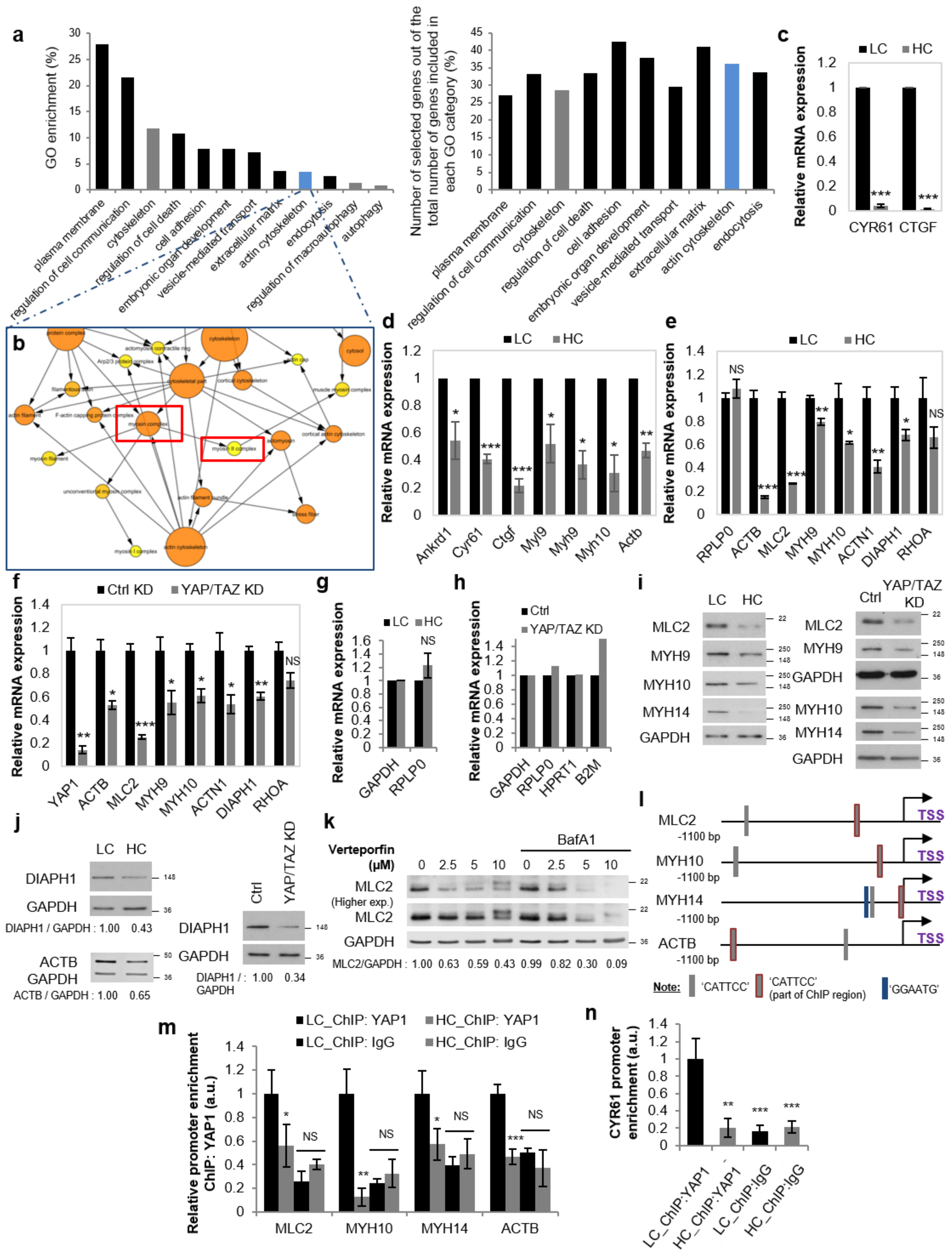

a
GO enrichment (%)
Number of selected genes out of the total number of genes included in each GO category (%)
plasma membrane
regulation of cell communication
cytoskeleton
regulation of cell death
cell adhesion
embryonic organ development
vesicle-mediated transport
extracellular matrix
actin cytoskeleton
endocytosis
regulation of macroautophagy
autophagy
b
myosin complex
myosin II complex
c
LC
HC
Relative mRNA expression
CYR61
CTGF
d
Ankrd1
Cyr61
Ctgf
Myl9
Myh9
Myh10
Actb
e
RPLP0
ACTB
MLC2
MYH9
MYH10
ACTN1
DIAPH1
RHOA
f
Ctrl KD
YAP/TAZ KD
YAP1
g
GAPDH
h
Ctrl
HPRT1
B2M
i
MYH14
j
DIAPH1 / GAPDH : 1.00 0.43
ACTB / GAPDH : 1.00 0.65
DIAPH1 / GAPDH : 1.00 0.34
k
Verteporfin (μM)
BafA1
0 2.5 5 10 0 2.5 5 10
MLC2 (Higher exp.)
MLC2/GAPDH : 1.00 0.63 0.59 0.43 0.99 0.82 0.30 0.09
l
-1100 bp
TSS
Note: 'CATTCC' 'CATTCC' (part of ChIP region) 'GGAATG'
m
LC_ChIP: YAP1
HC_ChIP: YAP1
LC_ChIP: IgG
HC_ChIP: IgG
Relative promoter enrichment ChIP: YAP1 (a.u.)
n
CYR61 promoter enrichment (a.u.)

Supplementary Fig. 6: Expression of actin related proteins is reduced in high confluency cells.

(a) Gene ontology analysis of genes previously identified as potential targets of YAP through ChIP-Seq. See Methods.

(b) Actin cytoskeleton gene ontology analysis for the selected potential targets (see (**a**)). Myosin complex and myosin-II form distinct groups.

(c) mRNA levels of CYR61 and CTGF relative to GAPDH in MCF10A cells plated at LC and HC. See also Fig. 2a. Bars – mean±s.e.m. (*n*=4; ****P*<0.001; two-tailed one sample t-test).

(d) mRNA levels of the indicated proteins relative to Rplp0 in primary MEFs at LC and HC. Bars – mean±s.e.m. (*n*=4; ****P*<0.001, ***P*<0.01, **P*<0.05; two-tailed one sample t-test).

(e-f) mRNA levels of the indicated proteins relative to GAPDH in HeLa cells **(e)** plated at LC and HC or **(f)** exposed to either control (Ctrl) or smart-pool YAP/TAZ siRNAs. Bars – mean±s.d. (*n*=3; ****P*<0.001, ***P*<0.01, **P*<0.05, NS – not significant; two-tailed t-test).

(g) mRNA levels of RPLP0 relative to GAPDH in MCF10A cells plated as in (**c**). Bars – mean±s.e.m. (*n*=4; ****P*<0.001; two-tailed one sample t-test).

(h) mRNA levels of the indicated proteins relative to GAPDH in HeLa cells exposed to YAP/TAZ siRNAs using the autophagy qPCR array (Qiagen).

(i) Representative western-blots for of the indicated proteins in MCF10A cells plated at different confluencies: LC or HC (left panel) and treated with YAP/TAZ siRNAs (right panel).

(j) Representative western-blots for the indicated proteins in MCF10A cells plated at various densities and exposed to YAP/TAZ siRNAs.

(k) Representative MLC2 immunoblot in HeLa cells exposed to increasing concentrations of verteporfin for 16 hours. MLC2 levels were quantified in the absence or presence of bafilomycin A1 (400 nM, 4 hours) relative to GAPDH, used as a loading control. MLC2 was revealed by ECL, while GAPDH was stained with LICOR secondary antibodies.

(l) Putative TEAD-binding sites in the promoter regions of the selected genes. The grey square corresponds to the 'CATTCC' motif, while the blue rectangular corresponds to the 'GGAATG' motif. The red line underlies the motifs included in the ChIP regions.

(m) Negative controls for Fig. 2e.

(n) Positive and negative controls for Fig. 2e. Bars – mean±s.d. (one representative experiment in triplicates; ****P*<0.001, ***P*<0.01; two-tailed t-test).

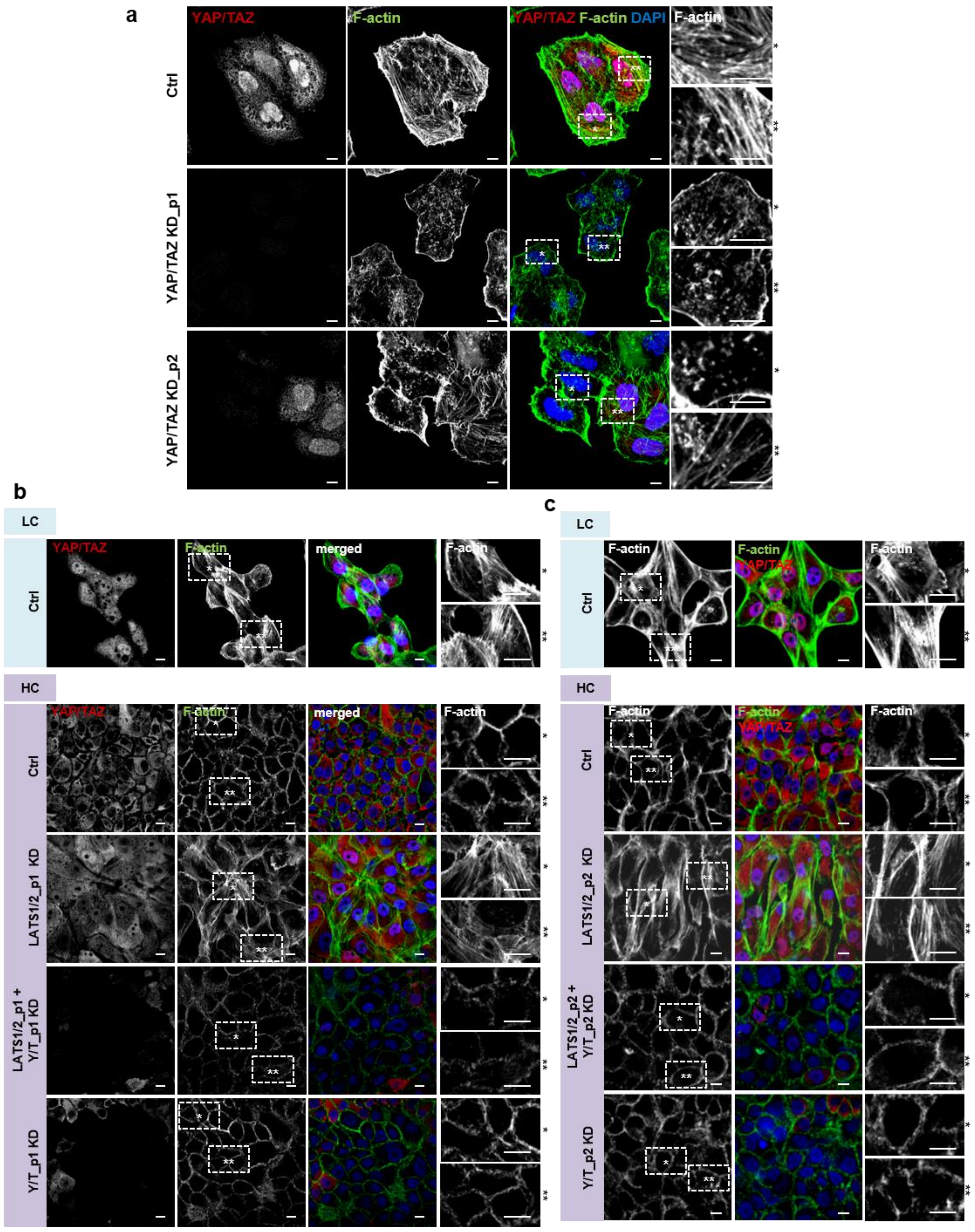
a
YAP/TAZ
F-actin
YAP/TAZ F-actin DAPI
F-actin
Ctrl
YAP/TAZ KD_p1
YAP/TAZ KD_p2
b
LC
HC
merged
LATS1/2_p1 KD
LATS1/2_p1 + Y/T_p1 KD
Y/T_p1 KD
c
LATS1/2_p2 KD
LATS1/2_p2 + Y/T_p2 KD
Y/T_p2 KD

Supplementary Fig. 7: YAP/TAZ knockdown reduces the amount of actin stress fibers.

(a) Representative confocal images of HeLa cells exposed to either control or two individual pairs of YAP + TAZ siRNA oligos (using two distinct pairs: either YAP/TAZ_p1 or YAP/TAZ_p2). HeLa cells were immunostained for endogenous YAP/TAZ and F-actin cytoskeleton (Phalloidin 488). Scale bars are 10 µm.

(b) Representative confocal images of MCF10A cells exposed to either control or individual pairs of LATS1 + LATS2 (LATS1/2_p1) and YAP + TAZ (Y/T_p1) siRNA oligos. MCF10A cells were immunostained for endogenous YAP/TAZ and F-actin. Scale bars are 10 µm. Distinct oligos were used in parallel experiment in (**c**).

(c) Representative confocal images of MCF10A cells exposed to either control or individual pairs of LATS1 + LATS2 (LATS1/2_p2) and YAP + TAZ (Y/T_p2) siRNA oligos. MCF10A cells were immunostained for endogenous YAP/TAZ and F-actin. Scale bars are 10 µm. Distinct oligos were used in parallel experiment in (**b**).

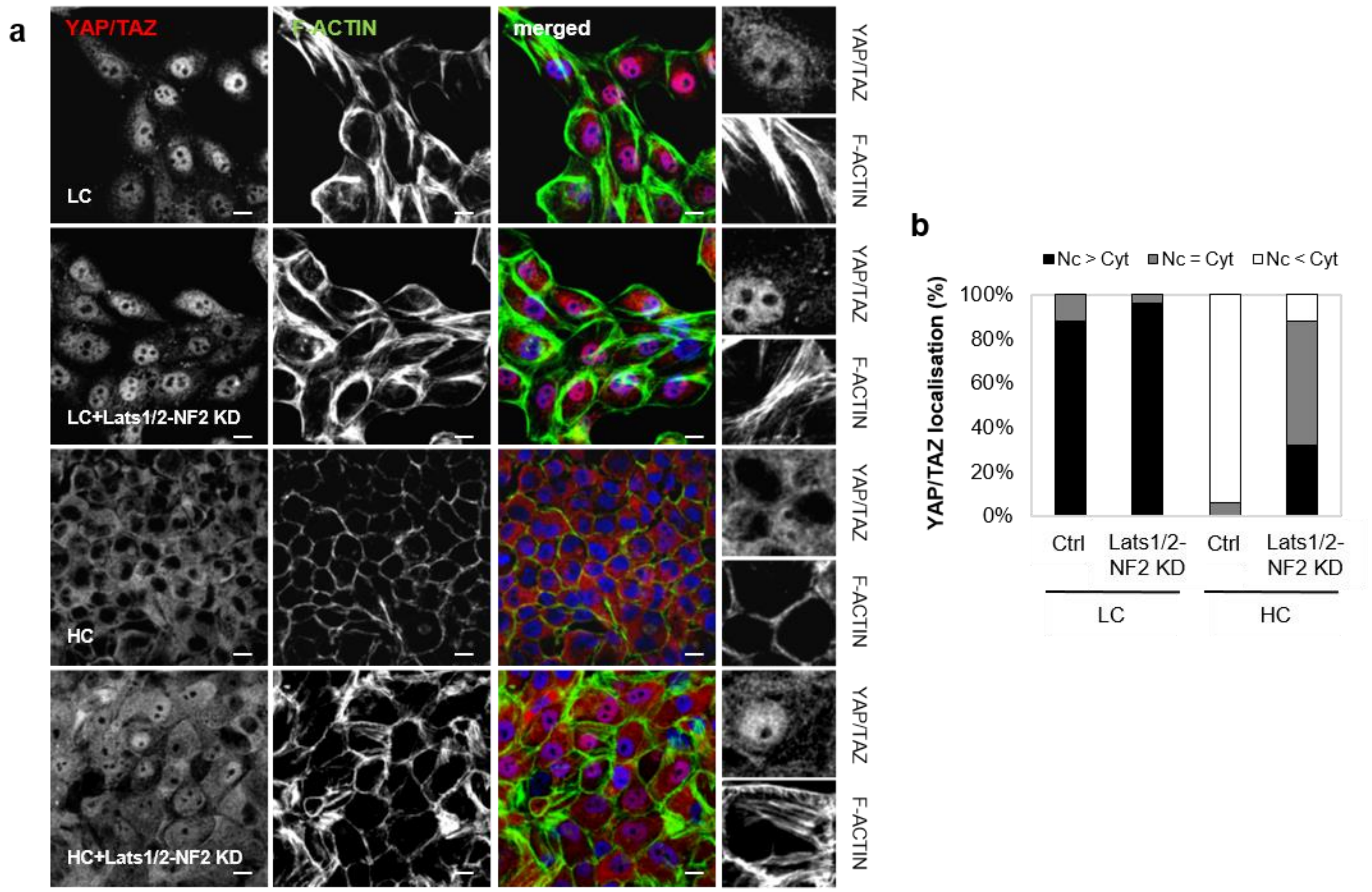

Supplementary Fig. 8: Double LATS1/2 and NF2 knockdown rescues YAP/TAZ localisation and F-actin stress fibers in MCF10A cells plated at HC..

(a) Representative confocal images of MCF10A cells exposed to either control or (LAST1/2 + NF2) siRNA oligos. MCF10A cells plated at various confluencies were immunostained for endogenous YAP/TAZ and F-actin. Scale bars are 10 µm.

(b) Percentage of MCF10A cells with either nuclear (Nc) or cytoplasmic (Cyt) YAP/TAZ localization. The cells were treated as in **(a)**.

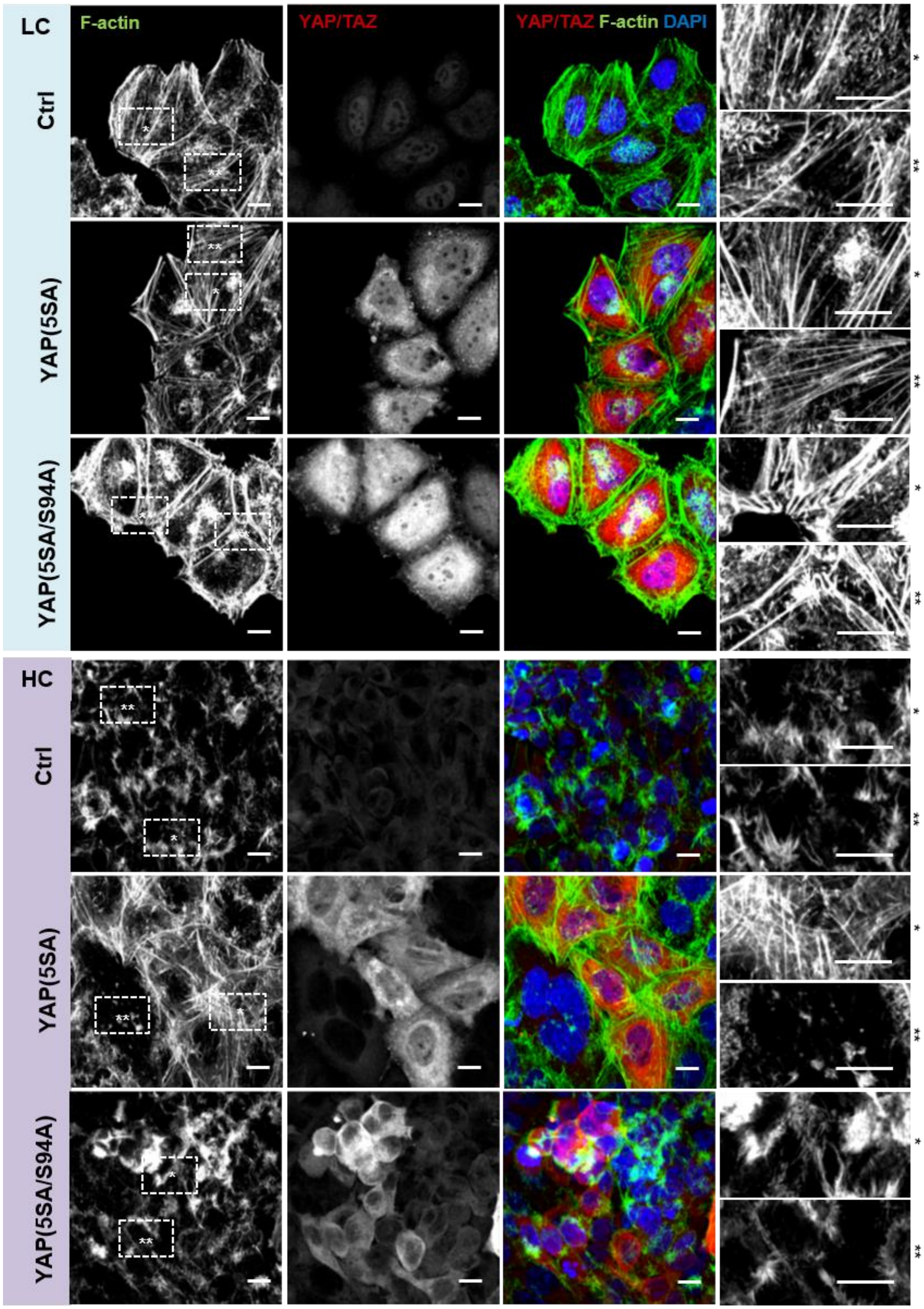

Supplementary Fig. 9: YAP(5SA) rescues the formation of actin stress fibers in high confluency HeLa cells

Representative confocal z-stacks of HeLa cells overexpressing control (empty flag), YAP(5SA) or YAP(5SA/S94A) were immunostained for endogenous YAP/TAZ and F-actin. Scale bars are 10 µm.

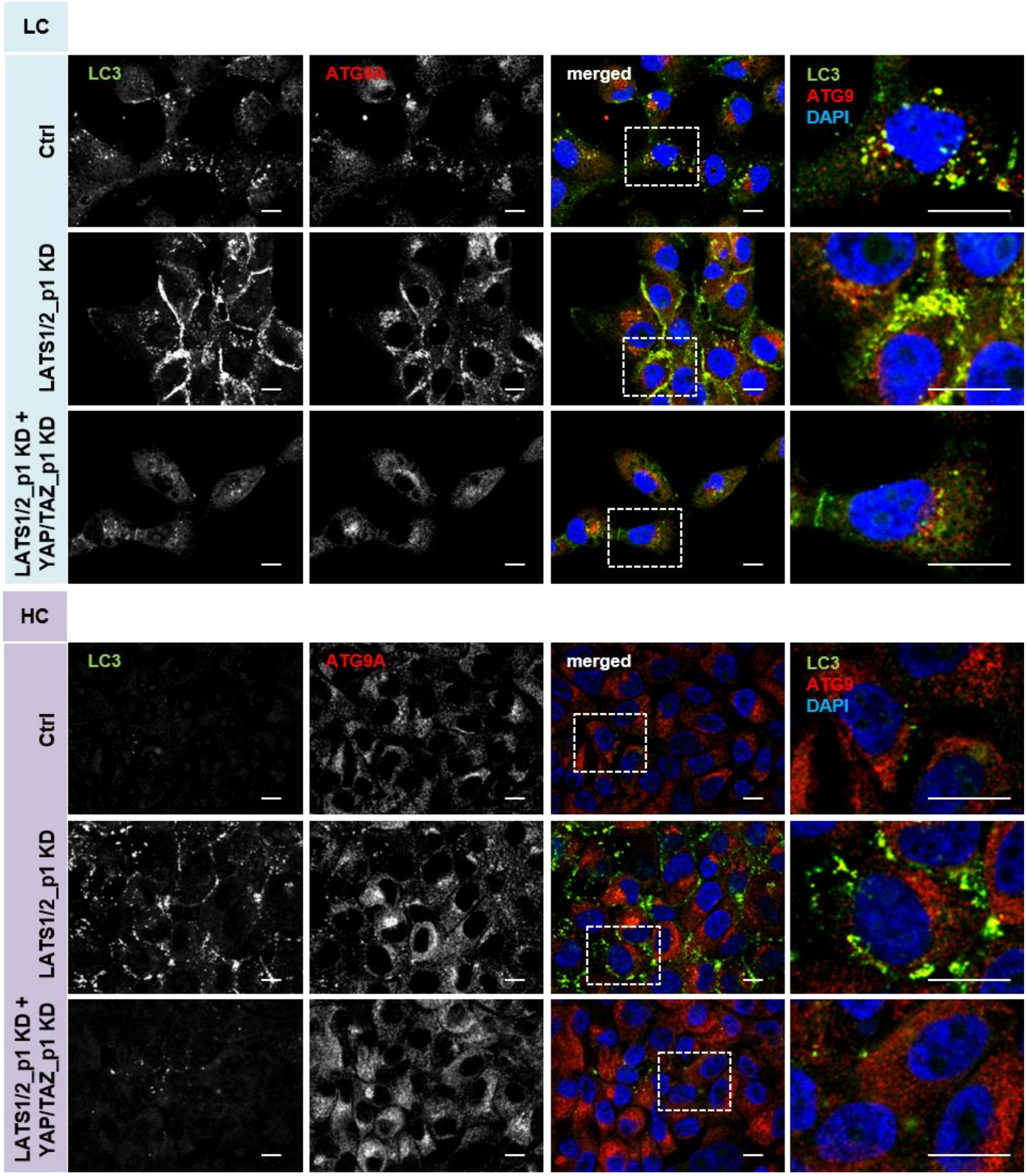

Supplementary Fig. 10: The decrease in ATG9A – LC3 colocalisation is YAP/TAZ-dependent in high confluency cells.

Representative images of LC3 and ATG9A double immunostaining in MCF10A cells plated at LC or HC confluencies and exposed to either LATS1/2_p1 alone or combined with YAP/TAZ_p1 siRNAs. Scale bar is 10 µm.

See Fig. 3e.

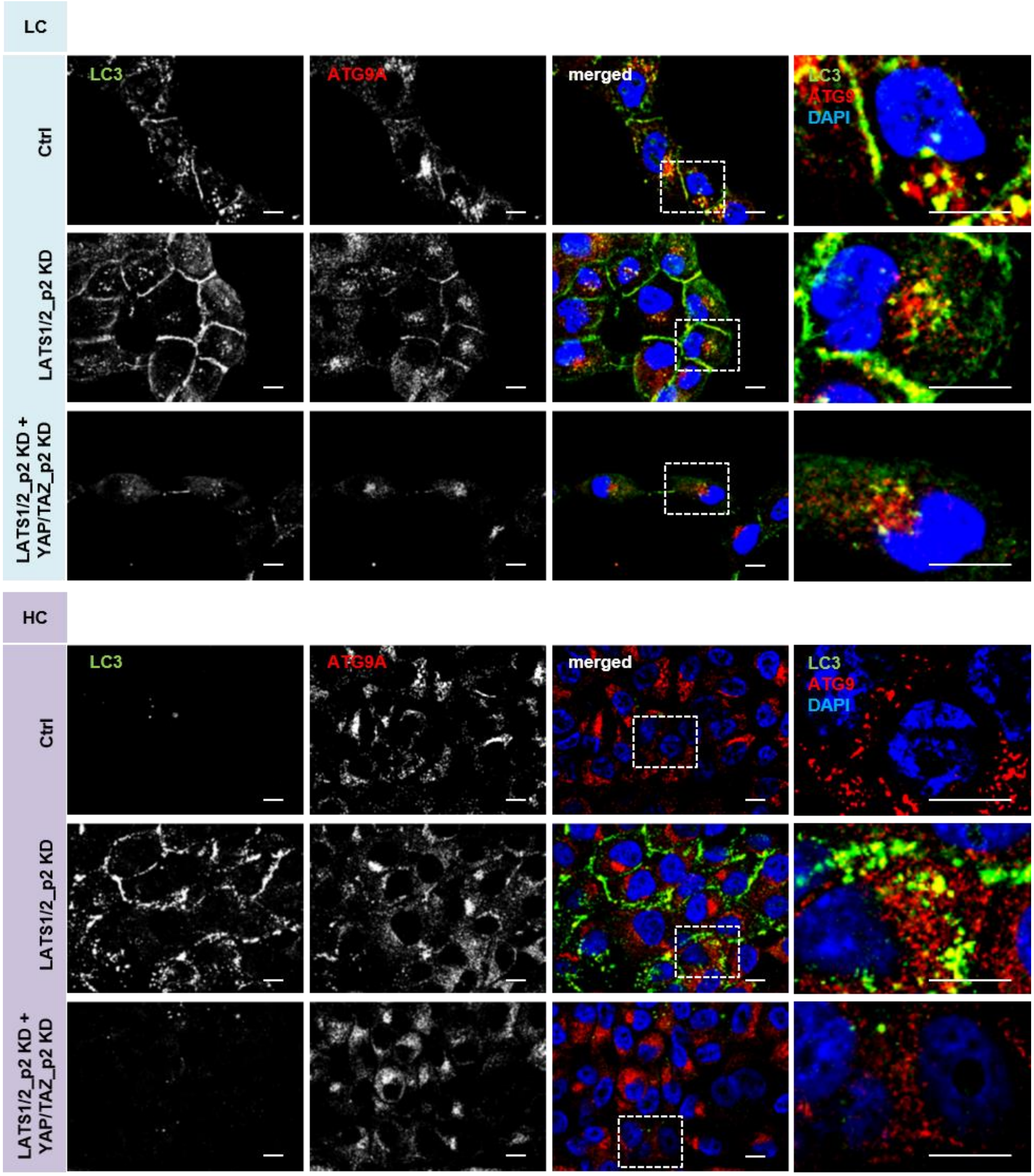

Supplementary Fig. 11: The decrease in ATG9A – LC3 colocalisation is YAP/TAZ-dependent in high confluency cells.

Representative images of LC3 and ATG9A double immunostaining in MCF10A cells plated at LC or HC confluencies and exposed to either LATS1/2_p2 alone or combined with YAP/TAZ_p2 siRNAs. Scale bar is 10 μm.

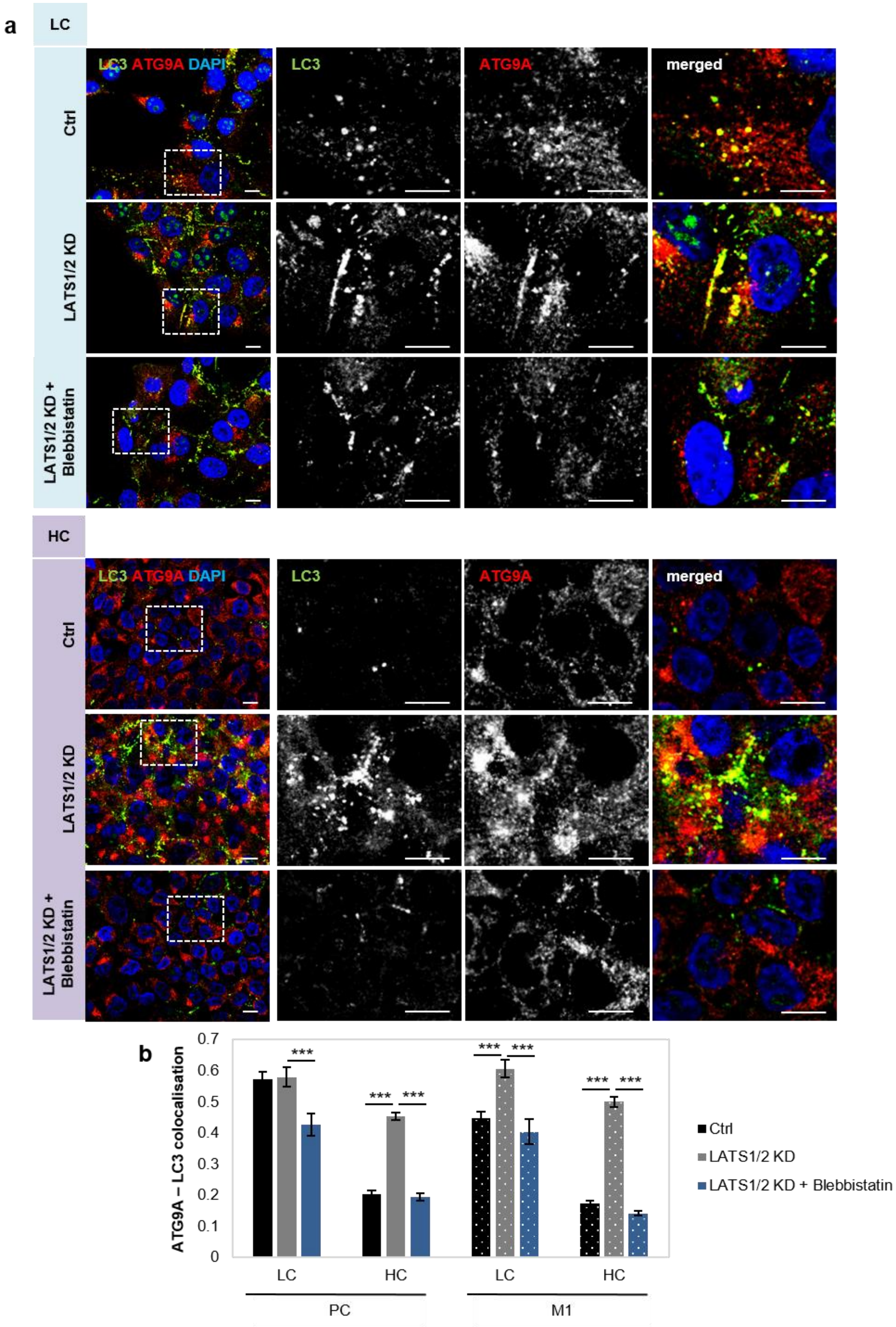
a
LC
HC
Ctrl
LATS1/2 KD
LATS1/2 KD + Blebbistatin
LC3 ATG9A DAPI
LC3
ATG9A
merged
b
ATG9A – LC3 colocalisation
0.7
0.6
0.5
0.4
0.3
0.2
0.1
0
***
LC
HC
PC
M1
Ctrl
LATS1/2 KD
LATS1/2 KD + Blebbistatin

Supplementary Fig. 12: The decrease in ATG9A – LC3 colocalisation is myosin-dependent in high confluency cells.

(a) Representative images of LC3 and ATG9A double immunostaining in MCF10A cells plates at LC and HC confluencies. MCF10A cells were transfected with either control or LATS1/2 siRNAs, plated at low and high confluencies and exposed to either DMSO (vehicle control) or blebbistatin (20 µM). Scale bar is 10 µm.

(b) ATG9A – LC3 colocalization in MCF10A cells plated and treated as in **(a)**. The Pearson's correlation and Manders' overlap (the amount of LC3 in the ATG9 compartment) coefficients were used to measure the colocalization of LC3 with ATG9A. Bars – mean±s.e.m. (for LC: n=25 cells, for HC: n=100 cells; ***P<0.001; two-tailed t-test).

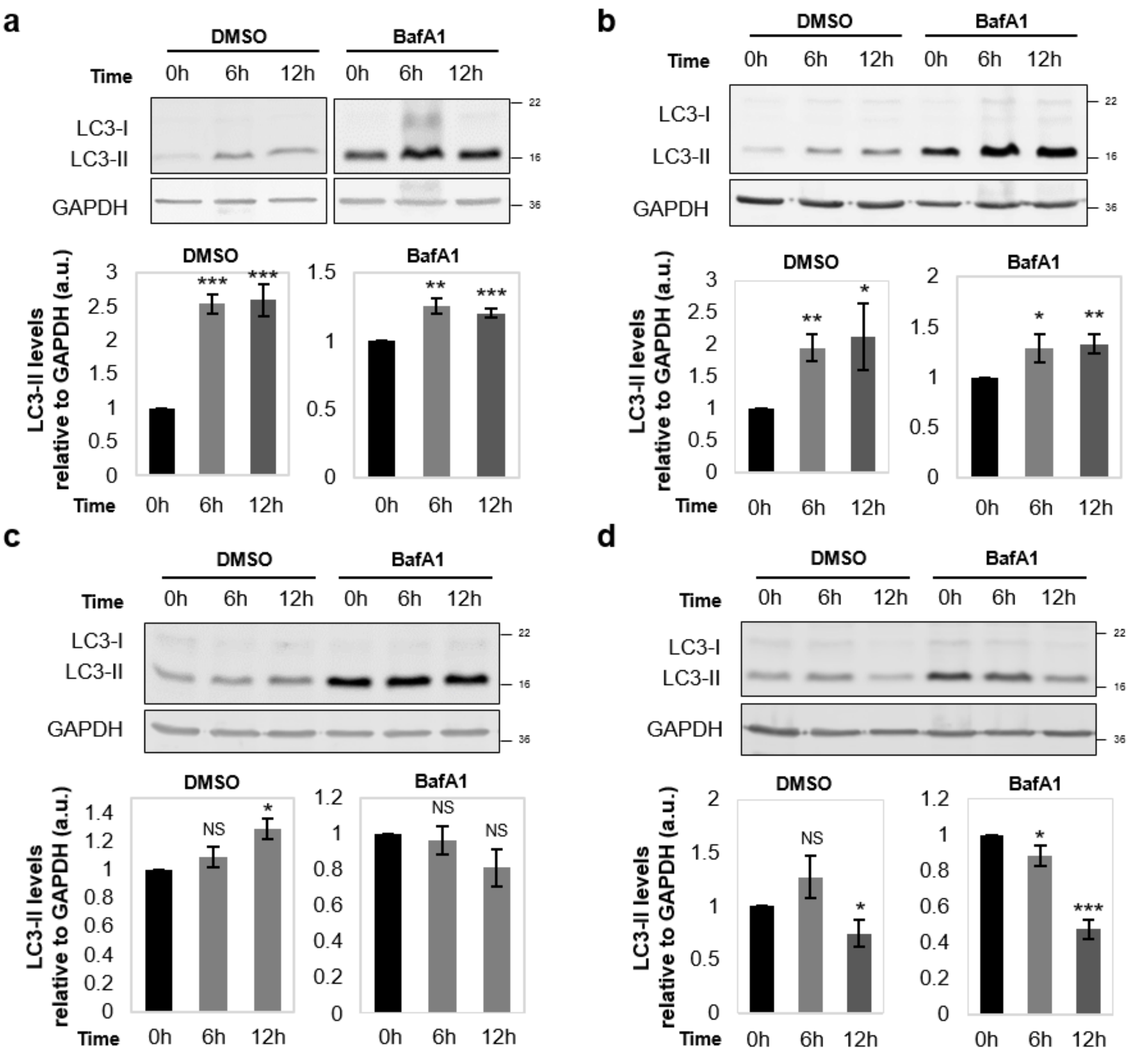

Supplementary Fig. 13: Cell suspension increases LC3-II levels only in MCF10A cells initially plated at LC (not HC).

(a) LC3-II levels assessed by immunoblotting in MCF10A cells plated at LC and then trypsinized and kept in suspension for various time points (6 and 12 hours). The graphs show the mean±s.d. (one representative experiment in triplicates; ***P<0.001, **P<0.01; two-way ANOVA). The experiment was repeated with similar results.

(b) LC3-II levels assessed by immunoblotting in HeLa cells plated at LC and then trypsinized and kept in suspension for various time points (6 and 12 hours). The graphs show the mean±s.d. (one representative experiment in triplicates; **P<0.01, *P<0.05; two-way ANOVA). The experiment was repeated with similar results.

(c) LC3-II levels assessed by immunoblotting in MCF10A cells plated at HC and then trypsinized and kept in suspension for various time points (6 and 12 hours). The graphs show the mean±s.d. (one representative experiment in triplicates; *P<0.05, NS – not significant; two-way ANOVA). The experiment was repeated with similar results.

(d) LC3-II levels assessed by immunoblotting in HeLa cells plated at LC and then scraped and kept in suspension for various time points (6 and 12 hours). The graphs show the mean±s.d. (one representative experiment in triplicates; *P<0.05, NS – not significant; two-way ANOVA). The experiment was repeated with similar results.

(a-d) The cells were treated with vehicle (DMSO) or bafilomycin A1 (BafA1) at 400 nM for the last 4 hours. GAPDH was used as loading control.

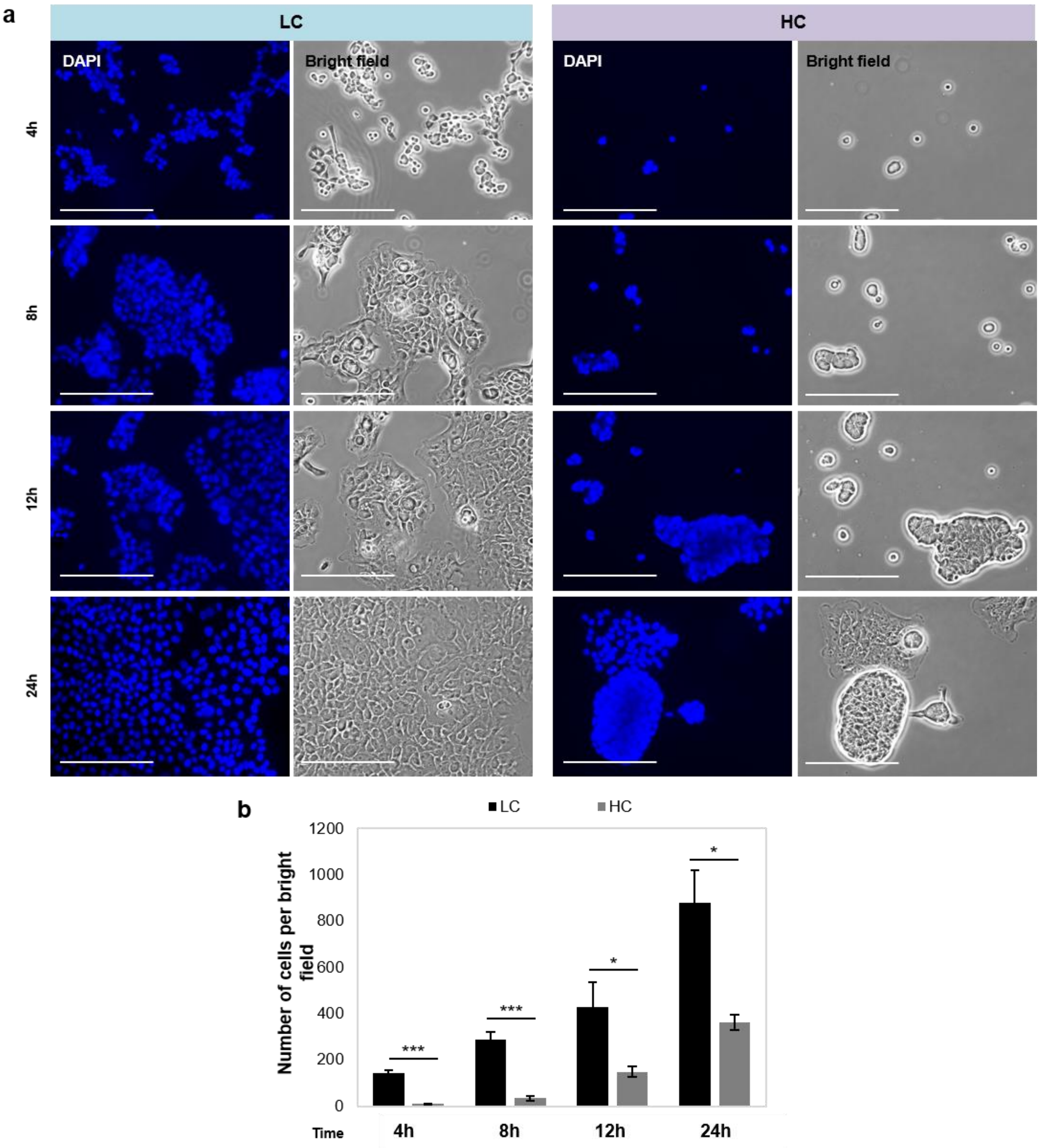

Supplementary Fig. 14: MCF10A cells plated at LC attach faster than those plated at HC.

(a) Representative DAPI/ Bright field images for MCF10A cell attachment. MCF10A cells were initially plated at LC and HC, then trypsinized and equal number of cells were seeded on coverslips and left to attach for 4, 8, 12 and 24 hours.

(b) Number of attached MCF10A cells per bright field at each indicated time point. The cells were treated as in **(a)**. The graphs show the mean±s.e.m. (n=4; ***P<0.001, *P<0.05; two-way ANOVA).

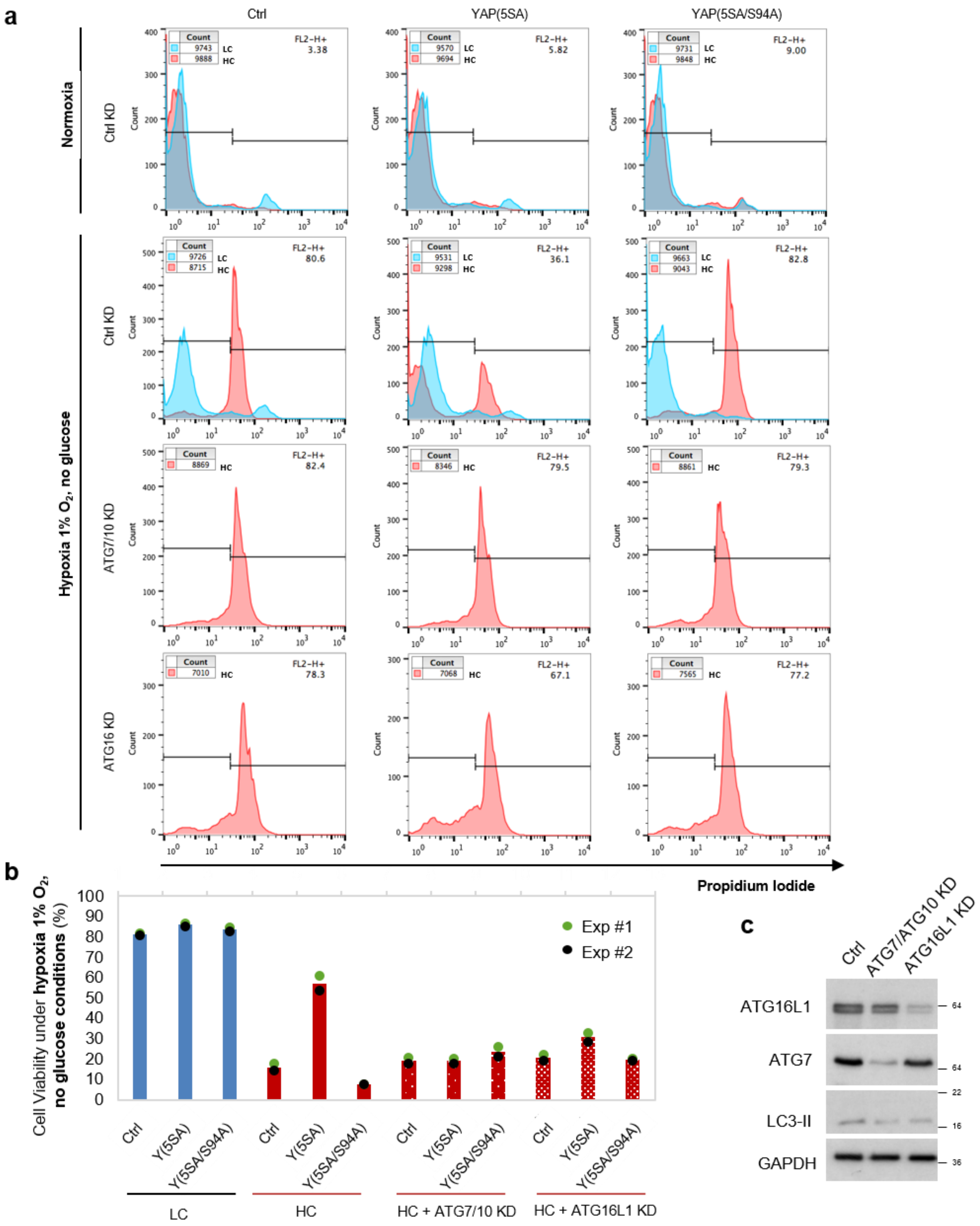

Supplementary Fig. 15: Cell viability is both YAP- and autophagy-dependent in high confluency HeLa cells under hypoxia and no glucose conditions.

(a) Propidium iodide (PI) staining and cell viability data measured by flow cytometry using the annexin5-FITC/PI method. HeLa cells exposed to control, ATG7/ATG10 or ATG16L1 siRNAs were transfected with control (empty flag vector), YAP(5SA) or YAP(5SA/S94A) and grown, for the last 16 hours, in hypoxia and no glucose conditions.

(b) Bars represent the mean of 2 independent experiments (Exp #1 – green dot-plot and Exp #2 – black dot-plot).

(c) The efficiency of autophagy gene knockdowns in HeLa cells.

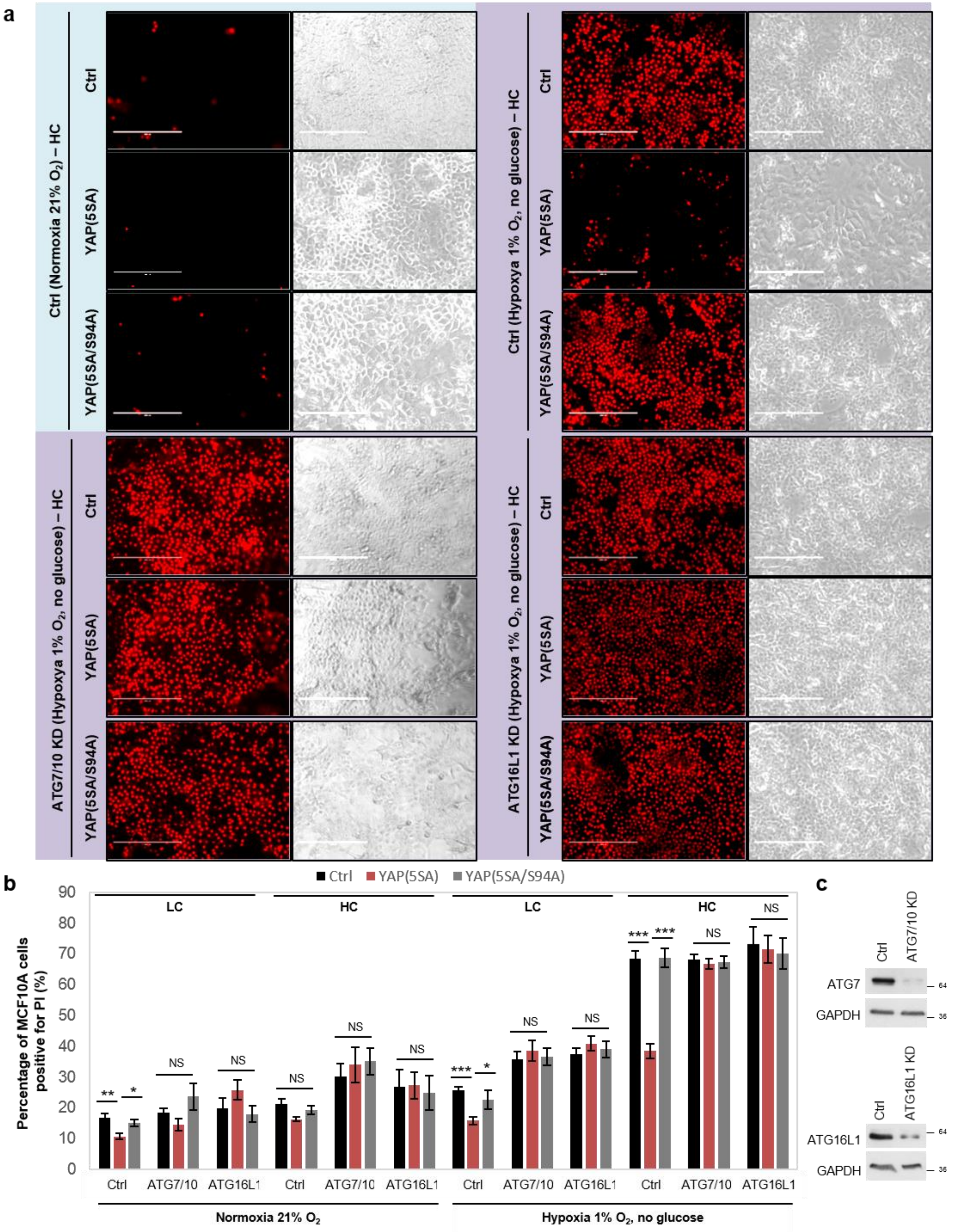

a
Ctrl (Normoxia 21% O2) – HC
Ctrl (Hypoxya 1% O2, no glucose) – HC
ATG7/10 KD (Hypoxya 1% O2, no glucose) – HC
ATG16L1 KD (Hypoxya 1% O2, no glucose) – HC
Ctrl
YAP(5SA)
YAP(5SA/S94A)
b
Ctrl
YAP(5SA)
YAP(5SA/S94A)
Percentage of MCF10A cells positive for PI (%)
LC
HC
NS
Normoxia 21% O2
Hypoxia 1% O2, no glucose
ATG7/10
ATG16L1
c
ATG7/10 KD
ATG16L1 KD
ATG7
ATG16L1
GAPDH
64
36

Supplementary Fig. 16: Cell viability is both YAP- and autophagy-dependent in high confluency MCF10A cells under hypoxia and no glucose conditions.

(a) Propidium iodide (PI) staining in MCF10A cells exposed to control, ATG7/ATG10 or ATG16L1 siRNAs and transfected with control (empty flag vector), YAP(5SA) or YAP(5SA/S94A). The cells were grown in hypoxia and no glucose conditions for the last 16 hours.

(b) Quantification of PI-positive cells by flow cytometry in MCF10A cells treated as in **(a)**. Bars represent the mean±s.e.m. (n=6; ***P<0.001, **P<0.01, *P<0.05, NS – not significant; two tailed t-test).

(c) The efficiency of the indicated autophagy gene knockdowns in MCF10A cells.

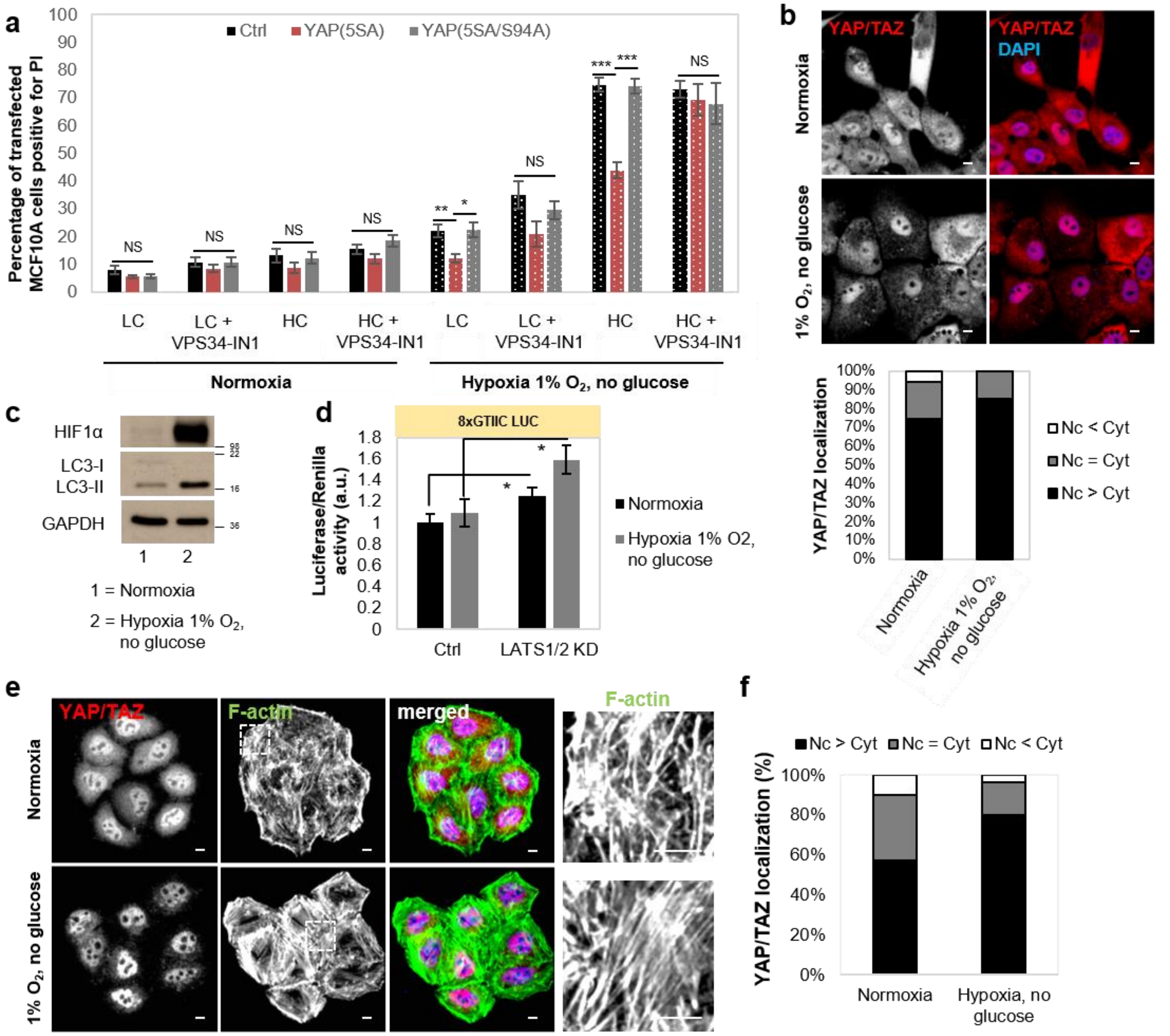

Supplementary Fig. 17: The apoptosis in high confluency cells is both YAP- and autophagy-dependent under hypoxia and glucose starvation conditions.

(a) MCF10A cells overexpressing control (empty flag vector), YAP(5SA) or YAP(5SA/S94A) were treated with either control (DMSO) or VPS34-IN1 and exposed to combined hypoxia and glucose-starvation conditions. These cells were stained with Propidium Iodide (PI) and the cell viability data was measured by flow cytometry. Bars represent the mean±s.e.m. (n=4; ***P<0.001, **P<0.01, *P<0.05, NS – not significant; two tailed t-test).

(b) YAP/TAZ localization in MCF10A cells plated at low confluency and grown under either normoxia or hypoxia + glucose starvation conditions. Percentages of cells with either nuclear or cytoplasmic localization are shown in the right panel: more than 400 cells were counted per condition.

(c) HIF1α immunoblot for the cells treated as in (**b**).

(d) Luciferase assay for YAP/TAZ activity in MCF10A cells transfected with either control or LATS1/2 siRNAs, plated at low confluency and grown under either normoxia or hypoxia + glucose starvation conditions.

(e) Representative images of YAP/TAZ and F-actin (Phalloidin 488) in HeLa cells plated at low confluency and grown under either normoxia or hypoxia and glucose starvation conditions. Percentages of cells with either nuclear or cytoplasmic localization are shown in the right panel: more than 400 cells were counted per condition. Scale bars throughout the panel are 10 µm.

(f) Quantification of YAP/TAZ localization for the HeLa cells treated as in (**e**).

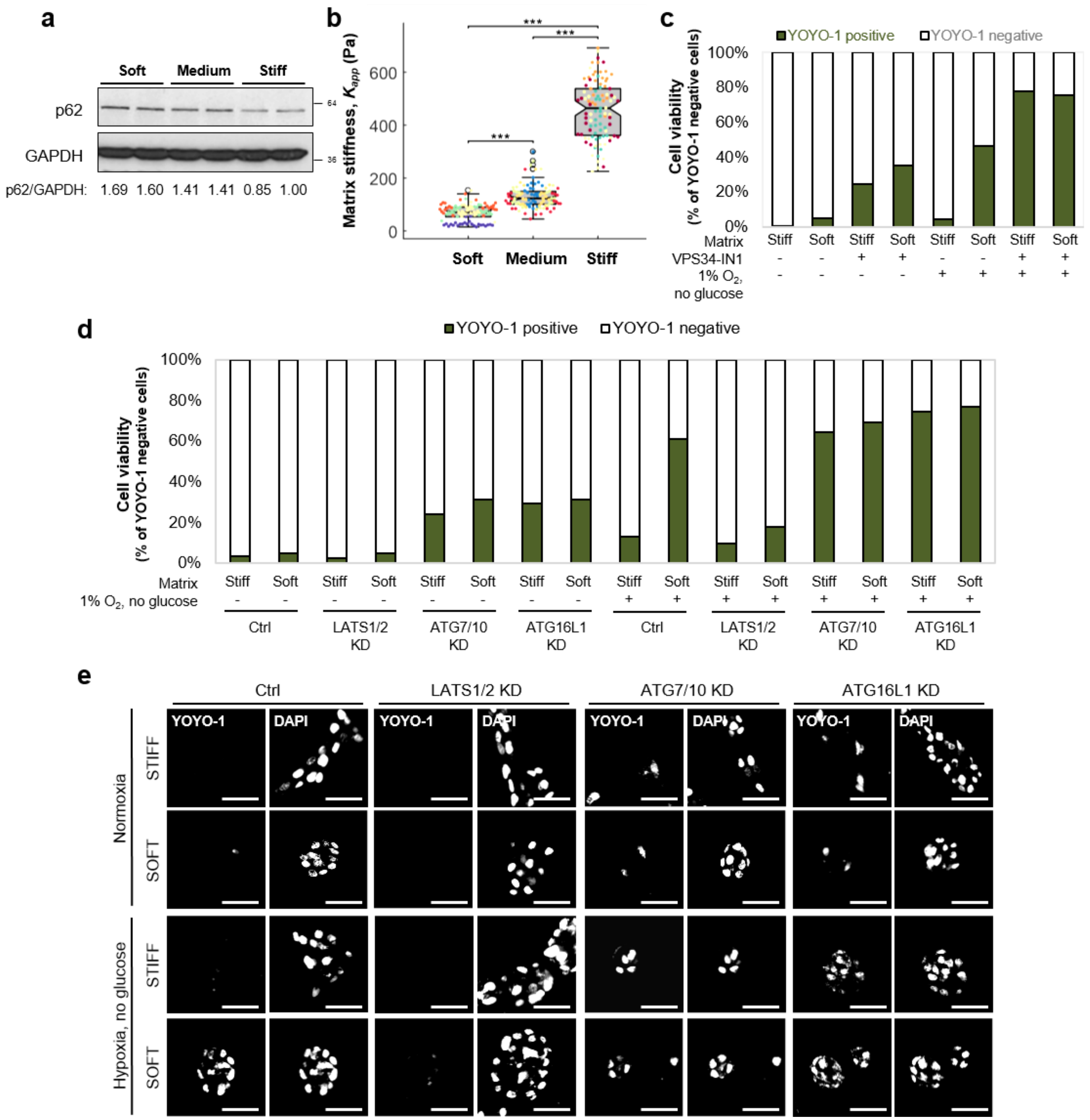

Supplementary Fig. 18: The apoptosis in soft matrix is both YAP- and autophagy-dependent under hypoxia and glucose starvation conditions.

(a) Representative p62 immmunoblot in MCF10A cells seeded on ECM with different stiffnesses.

(b) Apparent stiffness K_{app} of various collagen-based matrixes determined by AFM indentation experiments. See Methods.

(c) Quantification of YOYO-1 negative and positive MCF10A cells plated on stiff or soft ECM and exposed or not to hypoxia and glucose starvation (more than 200 cells were counted per each condition). The experiment was repeated with similar results.

(d) Quantification of YOYO-1 negative and positive MCF10A cells plated on stiff or soft ECM and exposed or not to hypoxia and glucose starvation (more than 200 cells were counted per each condition The cells were initially exposed to control, LATS1/2, ATG7/ATG10 or ATG16L1 siRNAs. The experiment was repeated with similar results.

(e) Representative images of YOYO-1 staining in MCF10A cells treated as in **(d)**. Scale bars throughout the panel are 50 µm.

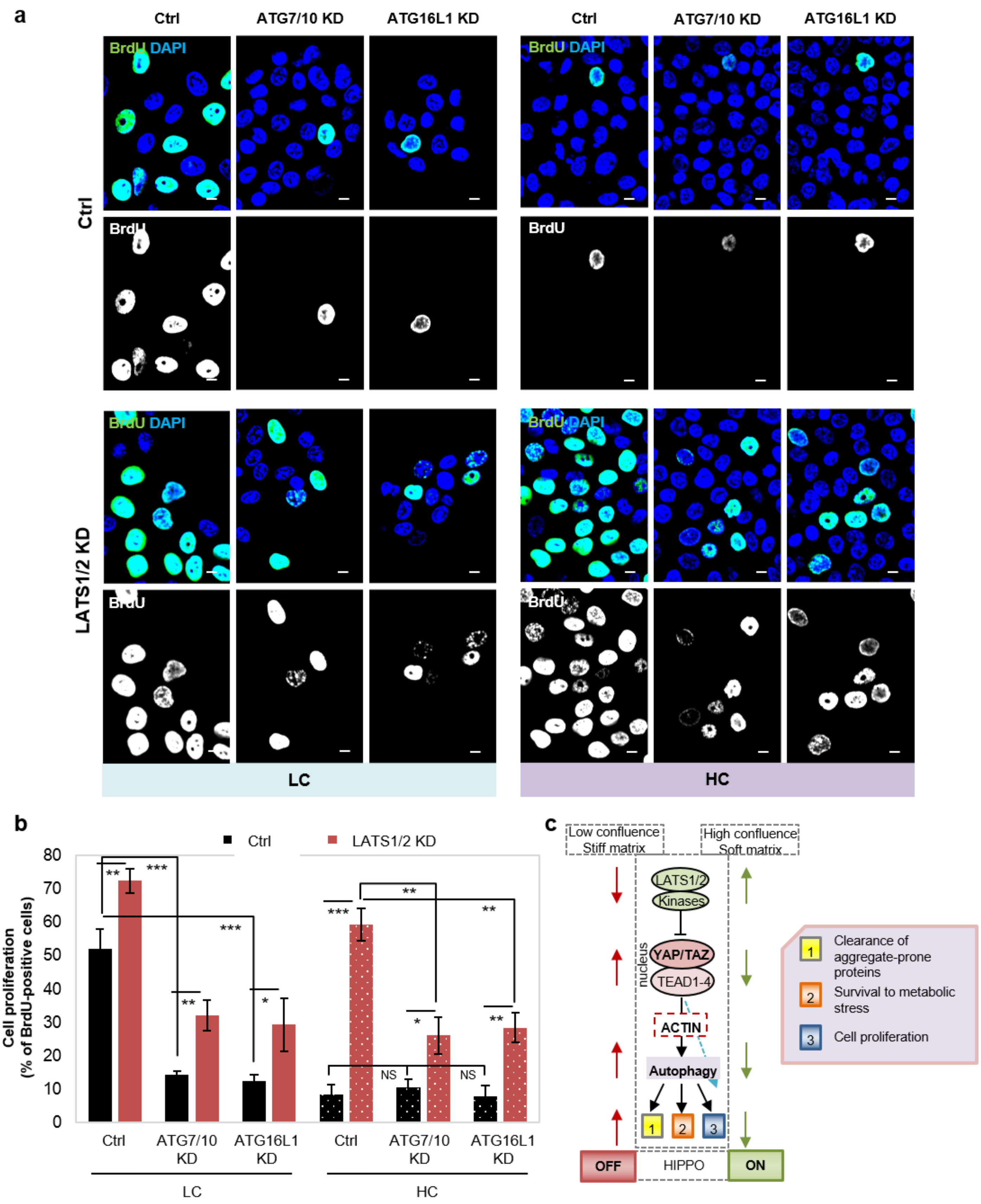

Supplementary Fig. 19: Autophagy controls cell proliferation downstream of LATS1/2 kinases.

(a) Representative BrdU images of MCF10A cells exposed to LATS1/2 siRNAs, plated at low and high densities. The cells were initially exposed to control, ATG7/ATG10 or ATG16L1 siRNAs. Scale bars are 10 µm.

(b) Quantification of BrdU-positive MCF10A cells treated as in **(a)**. Bars represent the mean±s.d. (n=3; ***P<0.001, **P<0.01, *P<0.05, NS – not significant; two-tailed t-test).

(c) Schematic representation of the roles of the YAP/TAZ-actomyosin-autophagy axis in the clearance of aggregate-prone proteins (such as polyQ-htt), survival to metabolic stress and cell proliferation.

Supplementary Fig. 20

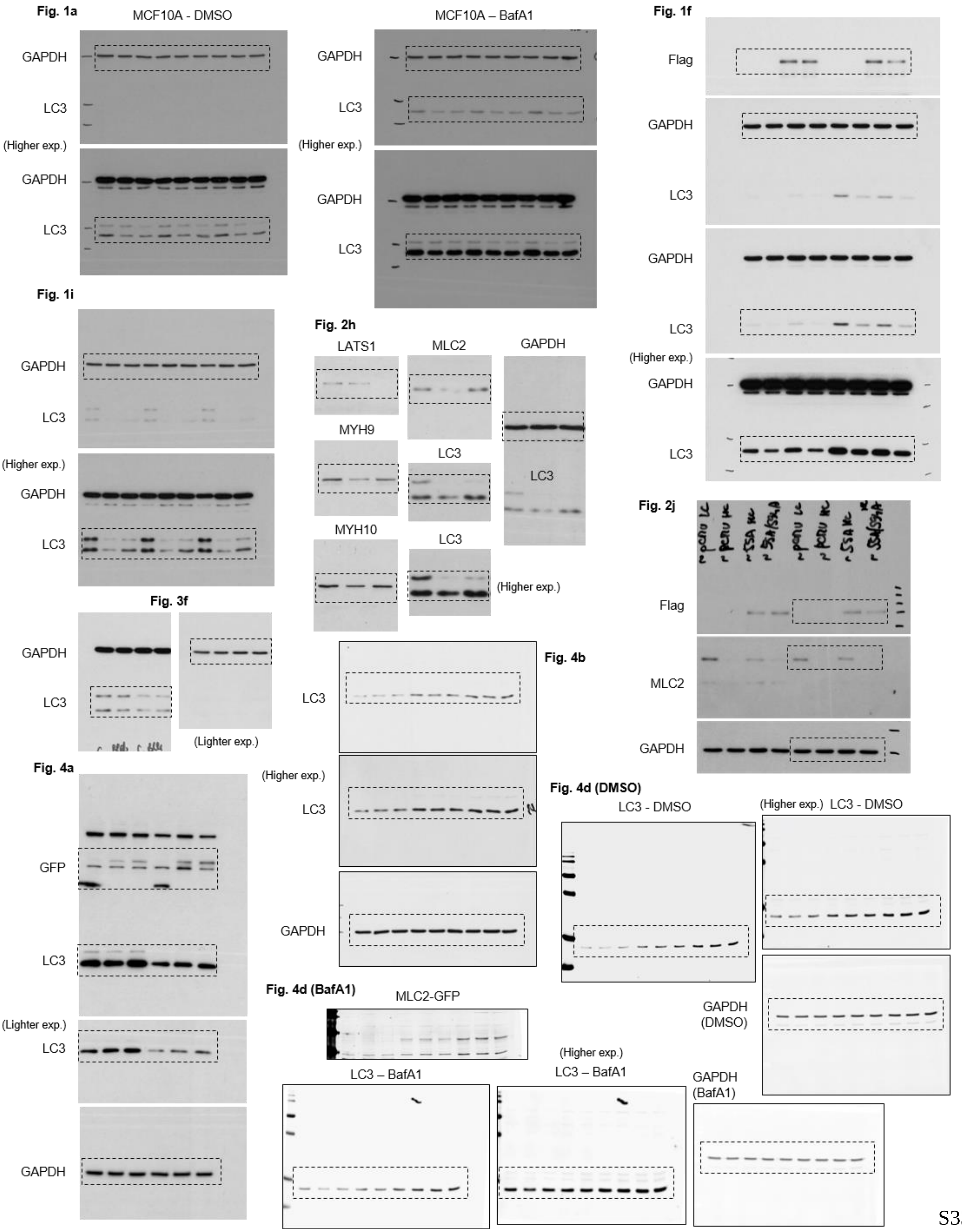

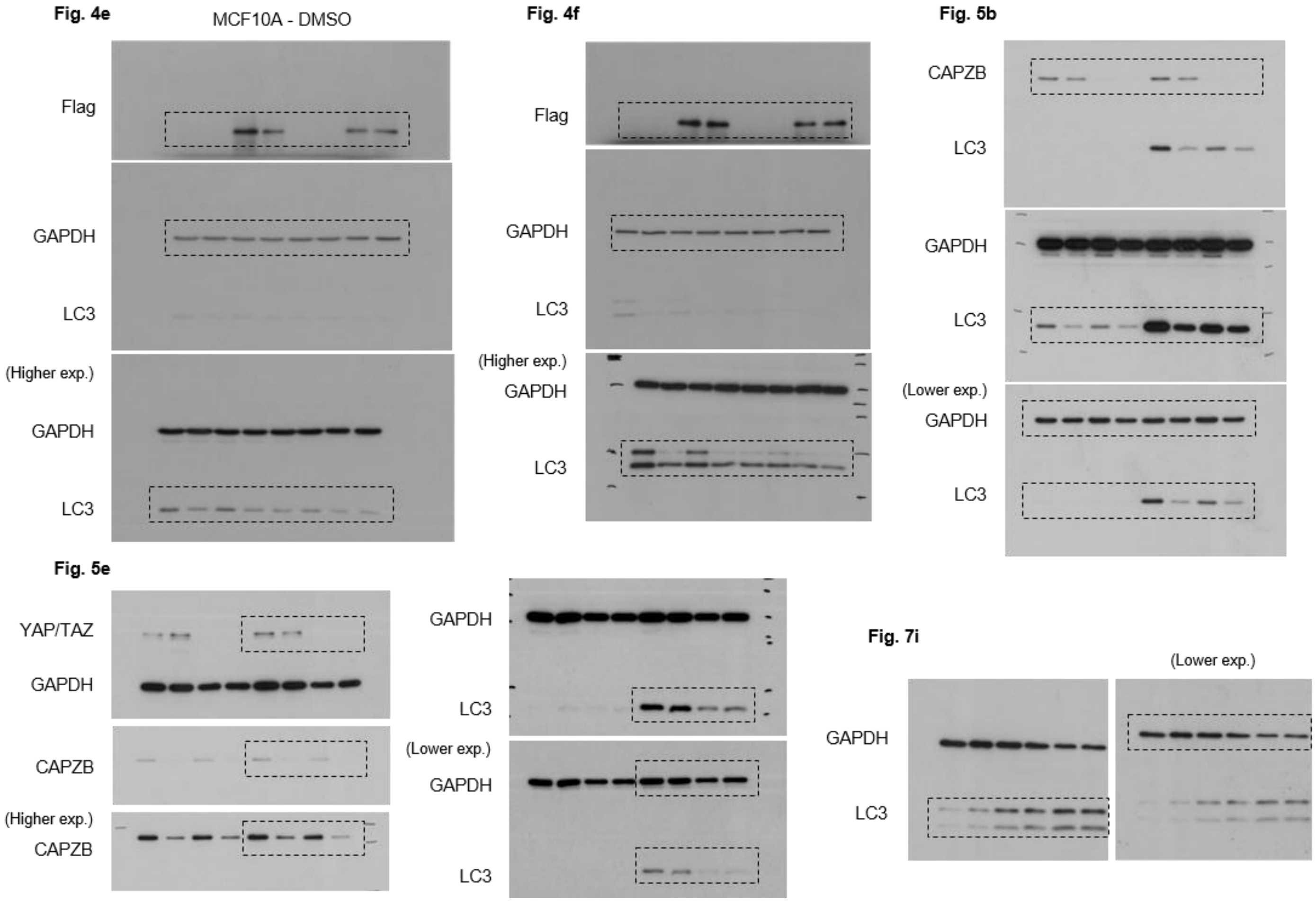

Supplementary Fig. 20. Full scans of uncropped blots.

Supplementary Table 1. Fold regulation of autophagy genes using RT^2 Profiler^{TM} PCR Array Human Autophagy (330231 QIAGEN) in YAP/TAZ–depleted compared to control HeLa cells.

Position	Symbol	Fold Regulation
G04	TGM2	-3.8107
C11	DRAM1	-2.6111
F02	NFKB1	-1.9656
B09	BCL2L1	-1.7385
F03	NPC1	-1.7326
D08	GABARAPL1	-1.7246
E12	MAPK8	-1.5434
E02	HSPA8	-1.428
A06	ATG16L1	-1.422
C06	CTSB	-1.4011
C01	CASP3	-1.363
B08	BCL2	-1.3424
A10	ATG4B	-1.3134
B02	ATG7	-1.3033
H01	ACTB	-1.2913
E11	MAPK14	-1.2802
B12	BNIP3	-1.2699
F11	RGS19	-1.2546
F06	PIK3R4	-1.2527
A07	ATG16L2	-1.2455
B05	BAD	-1.2451
B01	ATG5	-1.2397
D02	EIF4G1	-1.2363
G02	SQSTM1	-1.2289
F12	RPS6KB1	-1.2272
A08	ATG3	-1.2192
A11	ATG4C	-1.1948
D04	FADD	-1.1914
D12	HGS	-1.1887
E01	HSP90AA1	-1.187
B11	BID	-1.1822
A02	AMBRA1	-1.177
F08	PTEN	-1.175
E10	MAP1LC3B	-1.1633
F07	PRKAA1	-1.1457
A05	ATG12	-1.144
A03	APP	-1.1289
B06	BAK1	-1.1257
G11	UVRAG	-1.1139
A12	ATG4D	-1.11
C04	CDKN2A	-1.11
A01	AKT1	-1.1085
B10	BECN1	-1.1041
C12	DRAM2	-1.0973
F01	MTOR	-1.0768
E08	LAMP1	-1.0707
C02	CASP8	-1.068
A09	ATG4A	-1.0609
F04	PIK3C3	-1.0568
D09	GABARAPL2	-1.0528
D10	HDAC1	-1.0491
G12	WIPI1	-1.0007
D11	HDAC6	1.0277
E03	HTT	1.0376
C05	CLN3	1.0493
D06	GAA	1.0867
C03	CDKN1B	1.0942
E09	MAP1LC3A	1.0964
D05	FAS	1.102
B04	ATG9B	1.1075
C10	DAPK1	1.1075
D03	ESR1	1.1075
E04	IFNG	1.1075
E05	IGF1	1.1075
E06	INS	1.1075
E07	IRGM	1.1075
F05	PIK3CG	1.1075
G05	TMEM74	1.1075
G06	TNF	1.1075
G07	TNFSF10	1.1075
G10	ULK2	1.1075
B07	BAX	1.1141
C07	CTSD	1.1226
G08	TP53	1.1285
B03	ATG9A	1.135
F10	RB1	1.1687
D01	EIF2AK3	1.1715
G03	TGFB1	1.2674
F09	RAB24	1.2683
D07	GABARAP	1.3437
C09	CXCR4	1.3884
A04	ATG10	1.4063
C08	CTSS	1.642
G01	SNCA	1.9624
G09	ULK1	2.0837
H02	B2M	1.3602
H03	GAPDH	-1.1553
H04	HPRT1	-1.15
H05	RPLP0	-1.0238
H06	HGDC	1.1075
H07	RTC	1.2191
H08	RTC	1.1557
H09	RTC	1.1706
H10	PPC	1.256
H11	PPC	1.1915
H12	PPC	1.2861

Supplementary Table 2: Dharmacon siRNA sequences.

Target	Dharmacon identifier (smart pool)	Sequences
human ATG7	L-020112-00	J-020112-05: CCAACACACUCGAGUCUUU<br>J-020112-06: GAUCUAAAUCUCAAACUGA<br>J-020112-07: GCCCACAGAUGGAGUAGCA<br>J-020112-08: GCCAGAGGAUUCAACAUGA
human ATG10	L-019426-01	J-019426-09: CGUCUCAGGAUGAACGAAA<br>J-019426-10: AGGAAUUGCGGCACGAAGA<br>J-019426-11: GGAGGAGGCUUUCGAGCUA<br>J-019426-12: CCAACGUUAUUGUGCAGAA
human ATG16L1	L-021033-01	J-021033-09: UGUGGAUGAUUAUCGAUUA<br>J-021033-10: GGCACACACUCACGGGACA<br>J-021033-11: GCAUUGGAUUAACGGAAAC<br>J-021033-12: GUUAUUGAUCUCCGAACAA
human LATS1	L-004632-00	J-004632-05: GGUGAAGUCUGUCUAGCAA (LATS1_p1)<br>J-004632-06: UAGCAUGGAUUUCAGUAAU<br>J-004632-07: GGUAGUUCGUCUAUAUUAU (LATS_p2)<br>J-004632-08: GAAUGGUACUGGACAAACU
human LATS2	L-003865-00	J-003865-09: GCACGCAUUUUACGAAUUC (LATS2_p1)<br>J-003865-10: ACACUCACCUCGCCCAAUA<br>J-003865-11: AAUCAGAUAUUCCUUGUUG (LATS_p2)<br>J-003865-12: GAAGUGAACCGGCAAAUGC
human YAP1	L-012200-00	J-012200-05: GCACCUAUCACUCUCGAGA (YAP_p1)<br>J-012200-06: UGAGAACAAUGACGACCAA<br>J-012200-07: GGUCAGAGAUACUUCUUAA (YAP_p2)<br>J-012200-08: CCACCAAGCUAGAUAAAGA
human WWTR1 (TAZ)	L-016083-00	J-016083-05: CCGCAGGGCUCAUGAGUAU<br>J-016083-06: GGACAAACACCCAUGAACA (TAZ_p1)<br>J-016083-07: AGGAACAAACGUUGACUUA<br>J-016083-08: CCAAAUCUCGUGAUGAAUC (TAZ_p2)
Control (Non-targeting pool)	D-001810-10	Seq_1: UGGUUUACAUGUCGACUAA<br>Seq_2: UGGUUUACAUGUUGUGUGA<br>Seq_3: UGGUUUACAUGUUUUCUGA<br>Seq_4: UGGUUUACAUGUUUUCCUA

Supplementary Table 3: Primer sequences used for RT-qPCR.

Gene	Primers
human *ACTB*	Fw: 5’-GAGCACAGAGCCTCGCCTTT-3’<br>Rv: 5’-TCATCATCCATGGTGAGCTGG-3’
human *MLC2*	Fw: 5’-TTTGGGGAGAAGCTGAACGG-3’<br>Rv: 5’-TCATGGATGAAACCTGAGGC-3’
human *MYH9*	Fw: 5’-GGAAGGCTAAGCAAGGCTGA-3’<br>Rv: 5’-ACTTATAGCCAGGACCTGAACC-3’
human *MYH10*	Fw: 5’-GACTGAGGCGCTGGATCTGT-3’<br>Rv: 5’-CAAAAGCAATTGCCTCTTCAGC-3’
human *MYH14*	Fw: 5’-GCCCTGGAAGCCGACCAT-3’<br>Rv: 5’-GAAGGCACCCACACGAGAC-3’
human *ACTN1*	Fw: 5’-CACTTTGACCGGGATCACTCC-3’<br>Rv: 5’-TCATCCGTGTCCATCATGCC-3’
human *DIAPH1*	Fw: 5’-CGACGGCGGCAAATCTAAGAA-3’<br>Rv: 5’-CAGGTTCATATCCAGCAGCATC-3’
human *RHOA*	Fw: 5’-CGTTAGTCCACGGTCTGGTC-3’<br>Rv: 5’-GCCATTGCTCAGGCAACGAA-3’
human *RPLP0*	Fw: 5’-CGTCCTCGTGGAAGTGACAT-3’<br>Rv: 5’-TAGTTGGACTTCCAGGTCGC-3’
human *HPRT1*	Fw: 5’-CCTGGCGTCGTGATTAGTG-3’<br>Rv: 5’-TCAGTCCTGTCCATAATTAGTCC-3’
human *GAPDH*	Fw: 5’-ACAGTCAGCCGCATCTTCTTT-3’<br>Rv: 5’-CAATACGACCAAATCCGTTGACT-3’
human *CYR61*	Fw: 5’-AAGGAGCTGGGATTCGATGC-3’<br>Rv: 5’-CATTCCAAAAACAGGGAGCCG-3’
human *CTGF*	Fw: 5’-CACCCGGGTTACCAATGACA-3’<br>Rv: 5’-GGATGCACTTTTTGCCCTTCTTA-3’
human *YAP1*	Fw: 5’-CCCTCGTTTTGCCATGAACC-3’<br>Rv: 5’-GTTGCTGCTGGTTGGAGTTG-3’
mouse *Ankrd1*	Fw: 5’-GAGACACCCCACTGCATGAT-3’<br>Rv: 5’-TTCCCAGCACAGTTCTTGACC-3’
mouse *Cyr61*	Fw: 5’-AGAGGCTTCCTGTCTTTGGC-3’<br>Rv: 5’-CCAAGACGTGGTCTGAACGA-3’
mouse *Ctgf*	Fw: 5’-AGAACTGTGTACGGAGCGTG-3’<br>Rv: 5’-GTGCACCATCTTTGGCAGTG-3’
mouse *Myl9*	Fw: 5’-CTCTGCAGCAGGGAAACC-3’<br>Rv: 5’-CATGGCGAAGACATTGGACG-3’
mouse *Myh9*	Fw: 5’-AAGAAGGACCAGGGGGAGTT-3’<br>Rv: 5’-ATTCTTCACCGTCTTGGCGT-3’
mouse *Myh10*	Fw: 5’-TTCCTGGGGAACTTGAACGG-3’<br>Rv: 5’-CGGATAAACTTGCCAAAGCGA-3’
mouse *Actb*	Fw: 5’-TAGTTGGACTTCCAGGTCGC-3’<br>Rv: 5’-TCATCCATGGCGAACTGGTG-3’
mouse *Rplp0*	Fw: 5’-CGTCCTCGTTGGAGTGACAT-3’<br>Rv: 5’-TAGTTGGACTTCCAGGTCGC-3’
